# Dysregulation of the PATZ1/CTCF Balance Silences ZBTB20 to Drive Melanoma Progression

**DOI:** 10.1002/advs.202520917

**Published:** 2026-02-25

**Authors:** Chaowei Deng, Shuang Cai, Chen Guo, Shaker Khan, Lefan Liu, Qiong Tian, Zhiyuan Ma, Jian Zhang, Lingyu Zhao

**Affiliations:** ^1^ Institute of Genetics and Developmental Biology Translational Medicine Institute School of Basic Medical Sciences Xi'an Jiaotong University Health Science Center Xi'an Shaanxi China; ^2^ China Department of Cell Biology and Genetics/Key Laboratory of Environment and Genes Related to Diseases School of Basic Medical Sciences Xi'an Jiaotong University Health Science Center Xi'an Shaanxi China; ^3^ Department of Dermatology The First Affiliated Hospital of Xi'an Jiaotong University Xi'an Shaanxi China; ^4^ School of Stomatology Xi'an Jiaotong University Xi'an Shaanxi China

**Keywords:** chromatin architecture, CTCF, melanoma, PATZ1, ZBTB20

## Abstract

The transcription factor PATZ1 exhibits context‐dependent roles in human malignancies, yet its biological function and molecular mechanism in melanoma remain incompletely understood. Analysis of public databases and clinical specimens identifies significant PATZ1 overexpression in melanoma tissues, which strongly correlates with advanced disease stage and poor patient survival. Functional investigations demonstrate that PATZ1 drives melanoma cell proliferation, clonogenicity, migration, and invasion across melanoma genetic subtypes in vitro, while promoting tumor growth in vivo. Mechanistically, we discover that PATZ1 binds DNA via a conserved zinc finger domain to competitively displace the chromatin architectural protein CTCF from the promoter region of the tumor suppressor ZBTB20, thereby dysregulating their dynamic binding balance. This DNA‐binding‐dependent competition collapses a specific CTCF‐cohesion‐mediated chromatin loop, as directly demonstrated by chromosome conformation capture (3C) assays and validated through integrated multi‐omics data and functional enhancer deletion. Genetic rescue experiments confirm that ZBTB20 silencing is essential for PATZ1‐mediated oncogenicity. Furthermore, ZBTB20 transcriptionally represses PMEPA1 through direct promoter binding, thereby restraining the pro‐tumorigenic p38‐STAT1 signaling axis. Our findings define a complete PATZ1/CTCF‐ZBTB20‐PMEPA1‐p38‐STAT1 oncogenic pathway and establish that the dysregulation of the PATZ1/CTCF dynamic balance via DNA‐binding competition represents a novel epigenetic mechanism driving melanoma progression.

## Graphical abstract

This study uncovers a new oncogenic mechanism in melanoma. The transcription factor PATZ1 competes with the architectural protein CTCF for DNA binding, thereby disrupting a specific chromatin loop and silencing the tumor suppressor ZBTB20. This event unleashes the pro‐tumorigenic PMEPA1‐p38‐STAT1 signaling axis, promoting cancer progression. The work defines the complete “PATZ1/CTCF‐ZBTB20” pathogenic axis.

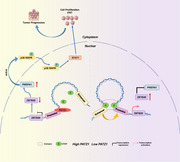

## 1 Background

Melanoma is a highly lethal form of skin cancer originating from melanocytes. Although it accounts for a small proportion of skin cancer cases, it is responsible for the vast majority of skin cancer‐related deaths [1]. Its incidence continues to rise globally [2], imposing a substantial disease burden [3]. Over the past decades, landmark therapeutic advances—particularly the introduction of immune checkpoint inhibitors and targeted agents such as BRAF/MEK inhibitors—have significantly improved patient outcomes [4, 5]. However, significant challenges remain: over half of metastatic patients succumb to the disease within 5 years due to therapy resistance [6, 7], highlighting the urgent clinical need to overcome resistance and develop novel treatment strategies. In this context, epigenetic regulatory mechanisms—which control gene expression through dynamic chromatin modifications without altering the DNA sequence—are increasingly recognized for their roles in melanoma pathogenesis, progression, and therapy resistance, emerging as a pivotal frontier for uncovering new therapeutic vulnerabilities and guiding innovative treatment approaches [8].

The dysregulation of epigenetic mechanisms, particularly the 3D architecture of the genome, is now recognized as a hallmark of cancer. At the heart of this architectural control is the precise spatial organization of the genome into topologically associating domains (TADs), which are critical for ensuring specific enhancer‐promoter interactions while preventing aberrant gene activation [9, 10, 11]. The CCCTC‐binding factor (CTCF), in conjunction with the cohesin complex (comprising SMC1, SMC3, and RAD21), is the master organizer of this chromatin topology [12, 13, 14, 15, 16, 17, 18, 19, 20, 21, 22]. CTCF binds to specific genomic sites, and through a process known as loop extrusion facilitated by cohesin, it anchors the formation of chromatin loops, thereby defining TAD boundaries [23, 24, 25]. This mechanism is essential for bringing distant regulatory elements, such as enhancers, into close proximity with their target promoters to control gene expression [1, 26, 27, 28, 29, 30, 31]. This CTCF‐mediated chromatin architecture is crucial for cell type‐specific gene expression programs during development and differentiation [32, 33]. In cancer, however, the function of this precise regulatory system is frequently subverted. Mutations or epimutations in CTCF binding sites, as well as alterations in CTCF or cohesin components themselves, can disrupt TAD integrity, leading to oncogene activation or tumor suppressor silencing—a phenomenon observed in gliomas, gastrointestinal stromal tumors, and other malignancies [34, 35]. While the consequences of such structural disruptions are increasingly documented, the upstream mechanisms that lead to CTCF dysfunction remain an area of active investigation. Although CTCF binding and function are known to be regulated by factors such as DNA methylation, histone modifications, and non‐coding RNAs [36, 37], the paradigm of a sequence‐specific transcription factor directly competing with CTCF for binding to its cognate sites, thereby subverting chromatin structure to drive tumor progression, represents a significant and underexplored gap in our understanding of cancer epigenetics. Consequently, identifying such trans‐acting competitors of CTCF represents a critical step toward understanding oncogenic rewiring.

Among the diverse family of transcription factors, the POZ/BTB domain‐containing proteins have emerged as critical regulators of cellular processes such as differentiation, proliferation, and tumor progression [38]. PATZ1 (POZ/BTB and AT hook containing zinc finger 1) is a key member of this family, functioning primarily as a transcriptional repressor by recruiting corepressor complexes (e.g., HDACs) to alter chromatin structure and inhibit gene expression [39, 40]. Its functional role is highly context‐dependent, acting as a documented oncogene in certain malignancies such as colorectal cancer by accelerating cell cycle progression and antagonizing p53 function [41, 42], while serving as a tumor suppressor in others, including thyroid cancer and glioblastoma, where it inhibits epithelial‐mesenchymal transition (EMT) and promotes apoptosis [43, 44]. This duality underscores the complex nature of PATZ1, whose activity is influenced by cell type, p53 status, and subcellular localization [45]. Given its role as a chromatin‐associated transcriptional repressor with oncogenic potential, we sought to investigate its undefined role in melanoma.

Our preliminary data firmly established PATZ1 as a bona fide oncoprotein in melanoma, evidenced by its significant overexpression correlating with poor patient prognosis and its potent tumor‐promoting functions in vitro and in vivo across genetic subtypes. Intriguingly, our initial exploration into its mechanism revealed, through motif analysis and genomic colocalization studies, that PATZ1 exhibits a high degree of similarity to, and co‐occupies chromatin sites with, the architectural protein CTCF. This critical observation led us to hypothesize that the oncogenic activity of PATZ1 in melanoma might be executed through a previously unrecognized mechanism: by competitively displacing CTCF from specific genomic loci, thereby disrupting the integrity of CTCF‐mediated chromatin loops and epigenetically silencing key tumor suppressor genes, ultimately driving tumor progression. This model posits a novel pathogenic mechanism wherein a transcription factor directly subverts genome architecture for oncogenic gain. To test this model and delineate the complete pathogenic axis, we designed a comprehensive experimental strategy. We first employed CUT&Tag and co‐immunoprecipitation assays to distinguish between competitive DNA binding and direct protein interaction. We then employed CUT&Tag to directly demonstrate the competitive interplay at the ZBTB20 locus, and identified ZBTB20 as a key transcriptional target subject to this PATZ1/CTCF competition. To establish causality and specificity, we generated a DNA‐binding‐deficient PATZ1 mutant and performed chromosome conformation capture (3C) assays to directly test the structural consequence of the competition. Subsequently, the specific enhancers involved in this ZBTB20 regulatory loop were identified and validated through an integrated approach, combining bioinformatic predictions, public Hi‐C data, and chromatin marks (H3K4me1, H3K27ac) with CRISPR/Cas9‐based functional deletion assays. Our investigation then extended beyond the epigenetic silencing event to mechanistically dissect how ZBTB20 loss contributes to tumor progression, leading us to uncover its role in restraining the PMEPA1‐p38‐STAT1 oncogenic signaling axis. The causal logic of the entire pathway, from PATZ1 expression to tumor phenotype, was cemented through a series of genetic rescue experiments. Furthermore, we conducted exploratory bioinformatic analyses to probe potential upstream drivers of PATZ1 overexpression itself.

In summary, this study not only defines the oncogenic role and a novel mechanism of action for PATZ1 in melanoma but also elucidates a broader pathogenic paradigm wherein a transcription factor promotes tumor progression by competitively disrupting the genome's higher‐order architecture. Our work thus establishes “architectural competition” as a distinct oncogenic process, providing new mechanistic insights and potential therapeutic vulnerabilities for this aggressive cancer.

## 2 Materials and Methods

### 2.1 Clinical Specimens

A total of eight patients with melanoma were enrolled in this study. Clinical tissue samples along with corresponding clinicopathological data, including age, sex, and initial tumor stage, were collected from all participants. All samples were procured from the Department of Surgical Oncology at the First Affiliated Hospital of Xi'an Jiaotong University. A critical inclusion criterion was that none of the patients had undergone any chemotherapy or radiotherapy prior to surgical resection, as explicitly documented in their pathological records. This study was conducted in accordance with the ethical standards and was approved by the Medical Ethics Committee of the First Affiliated Hospital of Xi'an Jiaotong University (LLSBPJ‐2023‐356).

### 2.2 Reagents and Antibodies

Antibodies PATZ1 (26225‐1‐AP) (RRID:AB_2880432), MMP2 (10373‐2‐AP) (RRID:AB_2250823), CDK2 (10122‐1‐AP) (RRID:AB_2078556), Cyclin D1 (26939‐1‐AP) (RRID:AB_2880691), and Beta‐Actin (66009‐1‐Ig) (RRID:AB_2687938) were purchased from Proteintech Group (Wuhan, China). Antibodies ZBTB20 (A7970) (RRID:AB_2772940), CTCF (A19588) (RRID:AB_2862685), and PMEPA1 (A12171) (RRID:AB_2759058) were purchased from Abclonal Technology (Wuhan, China). Antibodies p38 MAPK (T55600) (RRID:AB_2936971), Phospho‐p38 MAPK (Thr180/Tyr182) (TA4001) (RRID:AB_3712424), and STAT1 (T55227) (RRID:AB_2936355) were purchased from Abmart Inc. (Shanghai, China). Antibodies PATZ1(H‐2)X (sc‐393223 X) and ZBTB20(E‐11)X (sc‐515370 X) (RRID:AB_2924763) for CUT&Tag was purchased from Santa Cruz Biotechnology (Santa Cruz, CA).

### 2.3 Cell Culture

The human melanoma cell lines A375, MV3, A2058 (RRID:CVCL_1059), SK‐MEL‐28 (RRID:CVCL_0526), and SK‐MEL‐2along with the normal human melanocyte cell line PIG1, were utilized in this study. The human embryonic kidney 293‐FT cell line was used exclusively for lentiviral packaging. All cell lines were procured from Procell Life Science & Technology Co., Ltd. (Shanghai, China) in January 2023. Cells were cultured in a humidified incubator at 37°C with 5% CO_2_. Specifically, the A375, A2058, PIG1, and 293‐FT cell lines were maintained in high‐glucose Dulbecco's Modified Eagle Medium (DMEM), while the MV3, SK‐MEL‐28, and SK‐MEL‐2 cell lines were cultured in RPMI‐1640 medium. All media were supplemented with 10% fetal bovine serum (FBS) and 1% penicillin‐streptomycin solution. All cell lines were authenticated by short tandem repeat (STR) analysis and confirmed to be free of mycoplasma contamination prior to the commencement of the study. Mycoplasma testing was performed monthly using a PCR‐based assay, and all results were negative throughout the study period. The STR profiling report is available upon request.

### 2.4 Quantitative Real‐Time PCR (qRT‐PCR)

Total RNA was extracted from harvested cells and human tissue samples using TRIzol reagent (Invitrogen, Carlsbad, CA, USA) according to the manufacturer's protocol. As previously described [46]. The concentration and purity of the RNA were determined spectrophotometrically. Subsequently, 1 µg of total RNA was reverse‐transcribed into complementary DNA (cDNA) using the PrimeScript RT Reagent Kit (TaKaRa Biotechnology Co., Ltd., Dalian, China). Quantitative real‐time PCR was performed on an iCycler iQ Multicolor real‐time PCR Detection System (Bio‐Rad, Hercules, CA, USA) using a SYBR Green Premix Pro Taq HS qPCR Kit. The relative expression levels of target genes were normalized to the endogenous control GAPDH and calculated using the comparative 2^(‐ΔΔCt) method. Each sample was run in triplicate to ensure technical reproducibility and data reliability. All primers used were as follows:

PATZ1‐F: 5’‐Tgaaccagcagcgcaaaaac‐3’

PATZ1‐R: 5’‐Gagctgatagtgtgcatctcc‐3’

ZBTB20‐F: 5’‐Gacaggatctactcggcactc‐3’

ZBTB20‐R: 5’‐Actgcgccgctgtaaaaaga‐3’

PMEPA1‐F: 5’‐Tgtcaggcaacggaatccc‐3’

PMEPA1‐R: 5’‐Caggtacggataggtgggc‐3’

### 2.5 Transfection and Infection Experiments and Plasmids

Lentiviral vectors encoding short hairpin RNAs (shRNAs) against human PATZ1, as well as small interfering RNAs (siRNAs) targeting PATZ1, CTCF, and ZBTB20 (along with their respective negative controls), were procured from GenePharma Co., Ltd. (Shanghai, China) as follows:

Negative siRNA (NC‐siRNA) Sense: 5′‐UUCUCCGAACGUGUCACGUTT‐3′

Negative siRNA (NC‐siRNA) Antisense: 5′‐ACGUGACACGUUCGGAGAATT‐3′

siPATZ1#1 sense: 5′‐CGAGUACUUUGAGUCGGUGUU‐3′

siPATZ1#1 antisense: 5′‐AACACCGACUCAAAGUACUCG‐3′

siPATZ1#2 sense: 5′‐CGAGUACUUUGAGUCGGUGUU‐3′

siPATZ1#2 antisense: 5′‐AACACCGACUCAAAGUACUCG‐3′

siCTCF#1 sense: 5′‐CAAGAAUGAGAAGCGCUUUAA‐3′

siCTCF#1 antisense: 5′‐UUAAAGCGCUUCUCAUUCUUG‐3′

siCTCF#2 sense: 5′‐UAUGAUUUCCCAUCGACAUUU‐3′

siCTCF#2 antisense: 5′‐AAAUGUCGAUGGGAAAUCATA‐3′

siZBTB20#1 sense: 5′‐CCAGUGTAGUAUCUGCAACAA‐3′

siZBTB20#1 antisense: 5′‐UUGUUGCAGAUACUACACUGG‐3′

siZBTB20#2 sense: 5′‐CGCAGACAAACCAGCUAGAAA‐3′

siZBTB20#2 antisense: 5′‐UUUCUAGCUGGUUUGUCUGCG‐3′

sh‐Control: 5′‐AAAAGAGGCTTGCACAGTGCATTCAAGACGTGCACTGTGCAAGCCTCTTTT‐3′

sh‐PATZ1: 5′‐CCGGCGAGTACTTTGAGTCGGTGTTCTCGAGAACACCGACTCAAAGTACTCGTTTTTG‐3′

For overexpression, the full‐length coding sequences of human PATZ1, CTCF, and ZBTB20 were cloned into the PCDH‐CMV‐MCS‐EF1‐Hygro vector (Youbao Bio, Changsha, China). The plasmid for expressing the DNA‐binding deficient PATZ1 point mutant, PATZ1(C294A) (cysteine 294 to alanine), was generated and purchased from Genomeditech Co., Ltd. (Shanghai, China) in the same PCDH‐CMV‐MCS‐EF1‐Hygro backbone to ensure comparable expression. The successful construction and sequence of the mutant were confirmed by the vendor, and its loss of transcriptional repressor activity was validated in our laboratory (see Results). In this study, “PATZ1‐WT” refers to cells transfected with the wild‐type PATZ1 overexpression plasmid.

For transient transfection, A2058 and SK‐MEL‐28 cells were seeded in six‐well plates at a density of 2 × 10^5^ cells per well and transfected the following day using Lipofectamine 2000 (Invitrogen), with 2 µg of plasmid or a corresponding amount of siRNA per well; cells were harvested 72 h post‐transfection for analysis. For stable knockdown, A2058 cells at approximately 40% confluency were infected with lentiviral particles in two rounds, each lasting 12 h. Successfully infected cells were selected using 2 µg/mL puromycin (Life Technologies, New York, USA) for 72 h, and the resulting polyclonal populations were expanded and cryopreserved in liquid nitrogen for subsequent experiments.

### 2.6 CRISPR/Cas9‐Mediated Enhancer Deletion

To functionally validate the candidate enhancers upstream and downstream of the ZBTB20 promoter, we employed a dual‐sgRNA CRISPR/Cas9 strategy in the A2058 melanoma cell line to excise the entire core region of each enhancer. The pSpCas9(BB)‐2A‐Puro (PX459) V2.0 plasmid (Addgene plasmid #62988) was used for all editing experiments [47]. For each enhancer, two sgRNAs were designed to target the 5' and 3' boundaries, respectively, using the online tool CRISPOR (https://crispor.tefor.net/) with target sequences derived from the human reference genome GRCh38/hg38, as follows:

gRNA‐enhancer1‐ 5' boundaries‐sense: 5′‐ACCGACCAACTAGTTGGACTGTCA‐3′

gRNA‐enhancer1‐ 5' boundaries‐antisense: 5′‐AAACTGACAGTCCAACTAGTTGGTC‐3′

gRNA‐enhancer1‐ 3' boundaries‐sense: 5′‐CACCGCTAATTTGACCTAGGAAACC‐3′

gRNA‐enhancer1‐ 3' boundaries‐antisense: 5′‐AAACGGTTTCCTAGGTCAAATTAGC‐3′

gRNA‐enhancer2‐ 5' boundaries‐sense: 5′‐CACCGAACCAAATGTGGACCTCAGA‐3′

gRNA‐enhancer2‐ 5' boundaries‐antisense: 5′‐AAACTCTGAGGTCCACATTTGGTTC‐3′

gRNA‐enhancer2‐ 3' boundaries‐sense: 5′‐CACCGTATTTGTAGAAGACTATGTG‐3′

gRNA‐enhancer2‐ 3' boundaries‐antisense: 5′‐AAACCACATAGTCTTCTACAAATAC‐3′

Each sgRNA was individually cloned into the BbsI site of a separate PX459 plasmid. All constructs were verified by Sanger sequencing [48].

For the deletion of a specific enhancer, A2058 cells were co‐transfected with the corresponding pair of PX459 plasmids (each encoding one of the two boundary‐targeting sgRNAs) using Lipofectamine 2000. This co‐transfection strategy enables the simultaneous expression of both sgRNAs and the Cas9 protein in a single cell, facilitating the generation of two concurrent double‐strand breaks and the subsequent deletion of the intervening genomic region. 36 h post‐transfection, the culture medium was replaced, and cells were selected with 2 ug/mL puromycin for 72 h to enrich for cells that had taken up the plasmids. The polyclonal populations were then allowed to recover and expand.

As previously described [49], the successful excision of each enhancer region was confirmed by Sanger sequencing using primers flanking the predicted deletion boundaries, as follows:

Ontarget‐gRNA‐enhancer1‐ 5' boundaries‐sense: 5′‐TTTCTGTTCACAGCAGCCCT‐3′

Ontarget‐gRNA‐enhancer1‐ 5' boundaries‐antisense: 5′‐GGGTACAACCGGTGACACAA‐3′

Ontarget‐gRNA‐enhancer1‐ 3' boundaries‐sense: 5′‐TTGCAGCAGCTGAGGTAGTG‐3′

Ontarget‐gRNA‐enhancer1‐ 3' boundaries‐antisense: 5′‐TTTTACCTGCTCACCCTGCC‐3′

Ontarget‐gRNA‐enhancer2‐ 5' boundaries‐sense: 5′‐GTGTGAAGTCTGGGTGGCTT‐3′

Ontarget‐gRNA‐enhancer2‐ 5' boundaries‐antisense: 5′‐GCCAGGTGACATGCCATTTG‐3′

Ontarget‐gRNA‐enhancer2‐ 3' boundaries‐sense: 5′‐TCAGCTCAGGTCTCCTCTCC‐3′

Ontarget‐gRNA‐enhancer2‐ 3' boundaries‐antisense: 5′‐ACACCATATTCTGCGAGGGC‐3′

### 2.7 Western Blotting Assay

Total protein was extracted from melanoma cells using RIPA lysis buffer supplemented with PMSF and a protease inhibitor cocktail (Roche, Indianapolis, IN, USA). Nuclear protein extraction was performed using a commercial Nuclear Extraction Kit (SK‐0001, Signosis). Protein concentrations were determined using a BCA assay, and equal amounts of protein were separated by 10% SDS‐polyacrylamide gel electrophoresis and subsequently transferred onto polyvinylidene difluoride (PVDF) membranes. The membranes were blocked with 5% bovine serum albumin (BSA) for 2 h at room‐temperature and then incubated with specific primary antibodies overnight at 4°C. After washing, the membranes were incubated with corresponding horseradish peroxidase (HRP)‐conjugated secondary antibodies (anti‐mouse or anti‐rabbit) for 2 h at room‐temperature. Protein bands were visualized using a Super ECL detection reagent, and the chemiluminescent signals were captured and quantified using a Syngene GBox imaging system (Syngene, UK).

### 2.8 Co‐Immunoprecipitation (Co‐IP) Assay

To investigate a potential direct protein–protein interaction between PATZ1 and CTCF, co‐immunoprecipitation assays were performed using an epitope‐tagged overexpression system. Melanoma cells (A2058 or SK‐MEL‐28) were transiently transfected with a plasmid expressing full‐length human PATZ1 fused with a C‐terminal 3×Flag tag (PATZ1‐Flag) or with the corresponding empty vector as a negative control. Forty‐eight hours post‐transfection, cells were harvested and lysed on ice for 30 min in NP‐40 lysis buffer (50 mm Tris‐HCl, pH 8.0, 150 mm NaCl, 1% NP‐40) supplemented with a protease inhibitor cocktail. Cell lysates were clarified by centrifugation at 14000 × g for 15 min at 4°C.

For each immunoprecipitation reaction, 1 mg of total protein lysate was pre‐incubated with 1 µg of anti‐Flag antibody (Proteintech, 66008‐4‐Ig) for 2 h at 4°C with gentle rotation. Subsequently, 30 µL of pre‐washed Protein A/G Magnetic Beads (MCE, HY‐K0202) were added to each sample and incubated for an additional 2 h at 4°C to capture the antibody‐protein complexes. The beads were then collected using a magnetic stand and washed five times with cold lysis buffer. Bound proteins were eluted by boiling the beads in 2 × Laemmli SDS sample buffer for 10 min. The eluates (immunoprecipitates), along with 2% of the corresponding input lysates, were resolved by SDS‐PAGE and analyzed by Western blotting. Membranes were probed with an anti‐Flag antibody (Proteintech, 66008‐4‐Ig, 1:3000) to verify the immunoprecipitation of PATZ1‐Flag, and with an anti‐CTCF antibody (Abclonal, A19588, 1:1000) to assess the potential co‐precipitation of endogenous CTCF protein.

### 2.9 Cell Viability Detection

Cell viability was assessed using the MTT assay. Briefly, cells were seeded in 96‐well plates at a density of 3000 cells per well in 100 µL of the appropriate culture medium (DMEM or RPMI‐1640). After treatment with siRNA or overexpression plasmids for 24, 48, and 72 h, 10 µL of MTT solution (5 mg/mL) was added to each well, and the plates were incubated at 37°C for 4 h. Subsequently, the culture medium was carefully removed, and the formed formazan crystals were dissolved in 150 µL of dimethyl sulfoxide (DMSO). The absorbance of each well was measured at a wavelength of 492 nm using a POLARstar OPTIMA multifunctional microplate reader (BMG LabTechnologies, Germany). The relative cell viability was calculated by comparing the absorbance of treated groups to that of the control group.

### 2.10 Colony Formation Assay

After treatment with siRNA or overexpression plasmids for 6 h, cells were placed into six‐well plates at a density of 1500 cells/well and incubated for 14 days. After staining with 0.1% crystal violet for 30 min, the colonies were observed and quantified.

### 2.11 Cell Migration and Invasion Assay

Cell migratory and invasive capabilities were assessed using Transwell chambers (8.0 µm pore size; Corning Inc.). For the invasion assay, the chambers were pre‐coated with Matrigel, whereas for the migration assay, uncoated chambers were used. Briefly, cells resuspended in 200 µL of serum‐free medium were seeded into the upper chamber. The lower chamber was filled with 600 µL of complete medium containing 10% FBS as a chemoattractant. After incubation for 24 h at 37°C, the cells on the upper surface of the membrane were carefully removed with a cotton swab. The cells that had migrated or invaded to the lower surface were fixed with 4% paraformaldehyde for 30 min, stained with hematoxylin solution for 30 min, and then imaged under a microscope. The number of cells from five random fields per well was counted for quantitative analysis.

### 2.12 Cell Cycle Analysis

Cell cycle distribution was analyzed by flow cytometry. Briefly, treated melanoma cells were seeded in six‐well plates and harvested by trypsinization when they reached approximately 50% confluence within 24 h. The cells were collected, washed, and resuspended in phosphate‐buffered saline (PBS). For fixation, the cells were gently resuspended in ice‐cold 70% ethanol and incubated overnight at 4°C. Prior to analysis, the fixed cells were washed to remove ethanol and then stained with a solution containing 50 µg/mL propidium iodide (PI) and 50 µg/mL DNase‐free RNase A for 20 min at room‐temperature in the dark. Data acquisition was performed using a FACSCalibur flow cytometer (BD Biosciences, San Jose, CA, USA).

### 2.13 Apoptosis Analysis

Cell apoptosis was assessed using an Annexin V‐FITC/PI apoptosis detection kit. Treated melanoma cells were seeded in six‐well plates and allowed to reach approximately 50% confluence within 24 h prior to analysis. The cells were then harvested by trypsinization without EDTA, washed with cold PBS, and resuspended in 1X binding buffer at a density of 1 × 10^6^ cells/mL. According to the manufacturer's instructions, the cell suspension was stained with Annexin V‐FITC and propidium iodide (PI) for 15 min at room‐temperature in the dark. The stained cells were immediately analyzed using a FACSCalibur flow cytometer (BD Biosciences, San Jose, CA, USA).

### 2.14 CUT&Tag Assay and Data Processing

The CUT&Tag assay for PATZ1 and CTCF was performed using the Hyperactive Universal CUT&Tag Assay Kit for Illumina (Vazyme, TD903, Nanjing, China) following the manufacturer's protocol. Antibodies against PATZ1 and CTCF were used for chromatin immunoprecipitation. DNA was subsequently extracted and amplified using the i5 and i7 primers from the TruePrep Index Kit V2 for Illumina (Vazyme, TD202). The resulting libraries were purified with VAHTS DNA Clean Beads (Vazyme, N411) and sequenced on an Illumina platform by Novogene Co., Ltd. (Beijing, China) to generate 150 bp paired‐end reads.

For data processing, the quality‐controlled reads were aligned to the hg38 (Ensembl) human reference genome using Bowtie2 (v2.4.4) with the parameters –end‐to‐end –very‐sensitive –no‐mixed –no‐discordant. PCR duplicates were removed using Picard tools. Peak calling was conducted using MACS3 (v3.0.0), as previously described [50].

To quantitatively assess the binding of PATZ1 and CTCF at specific genomic loci (ZBTB20 promoter region), a CUT&Tag‐qPCR assay was performed. The chromatin immunoprecipitation procedure was identical to the CUT&Tag protocol described above, using antibodies against PATZ1 (Santa Cruz, sc‐393223 X) or CTCF (A19588). Following DNA extraction from the immunoprecipitated chromatin, the enrichment of specific genomic regions was quantified by quantitative real‐time PCR (qPCR) using SYBR Green chemistry on a Bio‐Rad iCycler iQ system. The relative enrichment was calculated using the comparative 2^(−ΔΔCt) method, normalized to the input DNA and expressed as fold change over the IgG control. The primers used for CUT&Tag‐qPCR to amplify the ZBTB20 promoter region were:

ZBTB20‐promoter‐F: 5‘‐AAGGTCAATCGTATCTCG‐3’

ZBTB20‐promoter‐R: 5‘‐TAAGCAACCTAACCTGAT‐3’

For the validation of ZBTB20 binding to the PMEPA1 promoter, a CUT&Tag‐qPCR assay was conducted. The procedure for chromatin immunoprecipitation using an anti‐ZBTB20 antibody was identical to the sequencing method described above. Following DNA extraction, the enrichment of specific genomic regions was quantified by quantitative real‐time PCR (qPCR) rather than by library preparation for sequencing. The relative enrichment was calculated as a fold change over the IgG control. The primers used for CUT&Tag‐qPCR to amplify the specific promoter region of PMEPA1 were designed as previously described [51].

### 2.15 Chromosome Conformation Capture (3C) Assay

The 3C assay was performed to quantitatively assess chromatin looping interactions between the ZBTB20 promoter and its candidate enhancer regions (Enhancer 1 and Enhancer 2). Briefly, approximately 1 × 10^7^ cells per sample were cross‐linked with 2% formaldehyde for 10 min at room‐temperature. Nuclei were isolated and lysed, and the chromatin was digested overnight with 300 units of EcoR I restriction enzyme. After digestion, chromatin fragments were ligated under dilute conditions using T4 DNA ligase at 16°C for 8 h to favor intra‐molecular ligation events. Following reversal of cross‐links and DNA purification, the relative interaction frequency was quantified by quantitative real‐time PCR (qPCR) using SYBR Green chemistry on a Bio‐Rad iCycler iQ system.

The 3C‐qPCR strategy employed a constant “anchor” forward primer designed within the ZBTB20 promoter region, paired with specific “target” reverse primers located within Enhancer 1 or Enhancer 2, respectively. A control genomic region with known, stable chromatin architecture was analyzed in parallel with a dedicated pair of primers to serve as an internal reference for data normalization and to control for variations in digestion and ligation efficiency across samples.

Primer sequences used for 3C‐qPCR:

ZBTB20 Promoter Anchor Primer (Forward): 5‘– ATGAGATTTATTATCCTGCCGGAA –3’

ZBTB20 Enhancer 1 Target Primer (Reverse): 5‘– ACCTCACCAAGCTCAGCCAC –3’

ZBTB20 Enhancer 2 Target Primer (Reverse): 5‘– AGCTTAGGCATTTCGGAGAAAGAC –3’

Control Region Primers:

Control Forward: 5‘– GGTGCCTATGGGTCAAAGTTT –3’

Control Reverse: 5‘– TCTATTTTCAGTCACTCAATCAGCC –3’

### 2.16 Subcutaneous Tumor Xenografts

All animal experiments were approved by the Biomedical Ethics Committee of Health Science Center of Xi'an Jiaotong University (Approval No. XJTUAE2024‐2373) and conducted in accordance with the National Institutes of Health Guide for the Care and Use of Laboratory Animals. This study was specifically carried out in compliance with the Guidelines for Animal Health and Use established by the Ministry of Science and Technology of China (2006). The maximal permitted tumor burden was 1500 mm^3^, and this limit was not exceeded in any of the animals during the study.

Twelve five‐week‐old female nude mice were purchased from Huafukang Biotechnology Co., Ltd. (Beijing, China) and randomly divided into two groups (*n* = 6 per group). After one week of acclimatization under specific pathogen‐free (SPF) conditions, the mice were anesthetized via isoflurane inhalation as previously described [52]. A2058 cells stably transfected with control shRNA (sh‐Ctrl) or PATZ1‐targeting shRNA (sh‐PATZ1) were harvested and resuspended in serum‐free RPMI‐1640 medium. Subsequently, 1 × 10^7^ cells in a 0.1 mL volume were subcutaneously injected into the left upper back of each mouse. The injection site was disinfected with 75% alcohol before and after the procedure to maintain aseptic conditions. Isoflurane anesthesia was employed for its rapid onset and controllability, with mice typically recovering within 2 min post‐anesthesia.

Tumor growth was monitored starting 7 days post‐inoculation. Tumor dimensions (length and width) were measured every two days with a digital caliper, and the volume was calculated using the formula: Volume = (length × width^2^) / 2. On day 24, all mice were humanely euthanized following isoflurane anesthesia. The subcutaneous tumors were carefully excised, weighed, and photographed next to a scale for size reference. Tumor tissues were then snap‐frozen in liquid nitrogen and stored at ‐80°C for subsequent analysis. No in vivo images of tumor‐bearing mice were captured, in strict adherence to journal policy.

### 2.17 Immunohistochemical (IHC) Staining

IHC staining was performed on 5 µm‐thick sections of paraffin‐embedded tumor tissues. The sections were first deparaffinized in xylene and rehydrated through a graded ethanol series. For antigen retrieval, the slides were heated in citrate buffer (pH 6.0) at 95°C for 20 min using a microwave oven. After cooling, endogenous peroxidase activity was quenched by incubation with 3% hydrogen peroxide, and non‐specific binding sites were blocked with 10% normal goat serum. The sections were then incubated overnight at 4°C with a primary antibody against PATZ1 (diluted 1:1000 in PBS). Following PBS washes, the sections were incubated with a horseradish peroxidase (HRP)‐conjugated secondary antibody at room‐temperature for 1 h. Immunoreactivity was visualized using a 3,3'‐diaminobenzidine (DAB) substrate kit, which produces a brown precipitate. Finally, the sections were counterstained with hematoxylin to provide nuclear contrast, dehydrated, cleared, and mounted for microscopic examination.

### 2.18 Luciferase Reporter Assay

To investigate the transcriptional regulation within the PATZ1/CTCF‐ZBTB20 axis, dual‐luciferase reporter assays were performed for both promoter and enhancer activities.

For promoter activity analysis, a DNA fragment encompassing the core promoter region of the human ZBTB20 gene was cloned into the pGL3‐Basic vector (Promega) to generate the ^*^pGL3‐ZBTB20^*^‐luc reporter plasmid. Melanoma cells (A2058 or SK‐MEL‐28) were co‐transfected with the ^*^pGL3‐ZBTB20^*^‐luc reporter plasmid (or the empty pGL3‐Basic vector as a negative control) and a Renilla luciferase plasmid (pRL‐TK, Promega) for normalization, along with either PATZ1/CTCF overexpression plasmids or their corresponding empty vectors.

For enhancer activity analysis, the genomic sequences identified as Enhancer 1 and Enhancer 2 were individually cloned into the pGL3‐Promoter vector (which contains a minimal SV40 promoter) upstream of the firefly luciferase gene, generating pGL3‐luc‐Enhancer1 and pGL3‐luc‐Enhancer2 constructs, respectively. These reporter plasmids were transfected into cells to assess their intrinsic transcriptional activation potential.

In all assays, 48 h post‐transfection, cells were harvested, and luciferase activities were measured using the Dual‐Luciferase Reporter Assay System (Promega) according to the manufacturer's instructions. Firefly luciferase activity was normalized to the Renilla luciferase activity in each sample. The relative activity was expressed as the fold change relative to the control group.

### 2.19 Analysis of Public Clinical Datasets and Bioinformatics Tools

To assess the clinical relevance and mechanistic underpinnings of our findings, we conducted comprehensive analyses using multiple public databases and bioinformatics tools.

#### 2.19.1 Clinical and Expression Analyses

The mRNA expression levels of PATZ1 and ZBTB20 in melanoma tissues compared to normal skin tissues were analyzed using the TCGA and GTEx cohorts via the Sangerbox platform (http://sangerbox.com/). The UALCAN portal (http://ualcan.path.uab.edu) was utilized to examine PATZ1 expression across disease stages (primary vs. metastatic) and to correlate ZBTB20 expression with clinical stage and nodal metastasis status. Patient survival analyses, including overall survival (OS), disease‐specific survival (DSS), and progression‐free interval (PFI), were performed based on TCGA data using the R2 Genomics Platform (https://hgserver1.amc.nl/cgi‐bin/r2/main.cgi) and Sangerbox. The R2 Genomics Platform utilizes a maximum‐statistic method (via log‐rank test scanning) to algorithmically determine the cutoff that best segregates patients into high‐ and low‐expression groups with the most significant survival difference. Gene expression correlation analyses (e.g., PATZ1 vs. ZBTB20, CTCF vs. ZBTB20) in melanoma samples were conducted using GEPIA2 (http://gepia2.cancer‐pku.cn/). The correlation between ZBTB20 and PMEPA1 mRNA expression was validated using melanoma cohorts from the cBioPortal (https://www.cbioportal.org).

#### 2.19.2 Mechanistic Bioinformatics Investigations

To generate mechanistic hypotheses, the DNA‐binding motif similarity between PATZ1 and CTCF was analyzed with the JASPAR database (https://jaspar.elixir.no). Genome‐wide colocalization analysis of PATZ1 and CTCF binding sites was performed using TFSyntax (https://tfsyntax.zhaopage.com/). To identify direct transcriptional targets of ZBTB20, we integrated our transcriptomic data with a public ZBTB20 ChIP‐seq dataset (ENCFF181BPM), which nominated PMEPA1 as a key candidate. Potential enhancer elements at the ZBTB20 locus were predicted using EnhancerAtlas (http://www.enhanceratlas.org). Furthermore, public Hi‐C data from multiple cell lines were integrated with our H3K4me1/H3K27ac CUT&Tag signals to define the chromatin architecture at this locus. Public ChIP‐seq datasets for cohesin subunits (SMC3: ENCFF029NYA; RAD21: ENCFF778YHU) and active promoter‐associated histone marks (H3K4me3: ENCFF269OOO, ENCFF006GYA; H3K9ac: ENCFF281TVA) were employed to validate binding and define the regulatory state at specific genomic loci. Data processing and visualization followed the default parameters of each respective platform.

#### 2.19.3 Analyses on the Upstream Regulation of PATZ1

To explore potential upstream drivers of PATZ1 overexpression in melanoma, we conducted the following bioinformatic analyses:

Analysis of Genetic Alterations: The cBioPortal platform was used to interrogate the TCGA Skin Cutaneous Melanoma (SKCM) cohort for copy‐number alterations (CNA) at the PATZ1 genomic locus. The correlation between PATZ1 copy‐number status and its mRNA expression (RNA‐seq V2 RSEM) was assessed using the built‐in tools of cBioPortal.

Analysis of Promoter DNA Methylation: The relationship between DNA methylation at the PATZ1 promoter region (specifically the CpG island spanning the transcription start site) and PATZ1 mRNA expression was analyzed using DNA methylation (Methylation 450K) data from the TCGA‐SKCM cohort accessed via cBioPortal.

Prediction and Correlation Analysis of Upstream Transcriptional Regulators: Potential transcription factors binding the PATZ1 promoter were predicted by integrating information from the JASPAR, GTRD, CHEA, KnockTF, ChIP_Atlas, hTFtarget, and ENCODE databases (https://jingle.shinyapps.io/TF_Target_Finder/). The expression correlations between candidate regulators (STAT3, MITF) and PATZ1 in melanoma patient samples were evaluated using the cBioPortal correlation analysis module based on TCGA data.

### 2.20 Statistical Analysis

Data are presented as the mean ± standard deviation (SD). No specific data transformation was applied, and no outliers were excluded from the analyses unless otherwise stated. The sample size (n) represents the number of independent biological replicates (e.g., independently performed experiments on different days with distinct cell passages) and is indicated in the respective figure legends.

Statistical analyses were performed using GraphPad Prism software (version 6.0). For comparisons between two groups, an unpaired, two‐tailed Student's *t*‐test was used after confirming the assumption of equal variances with an F‐test. For comparisons among multiple groups, one‐way analysis of variance (ANOVA) or two‐way ANOVA was applied as appropriate, followed by Tukey's post‐hoc test for multiple comparisons. Correlation analyses between gene expression levels in public cohorts were evaluated using Pearson's correlation coefficient. The threshold for statistical significance was set at a *p*‐value (alpha) of less than 0.05. Significance levels are denoted as follows: ns, not significant; ^*^ *p* < 0.05; ^**^ *p* < 0.01; ^***^ *p* < 0.001.

## 3 Results

### 3.1 PATZ1 is Overexpressed in Melanoma and Predicts Poor Patient Survival

**FIGURE 1:**
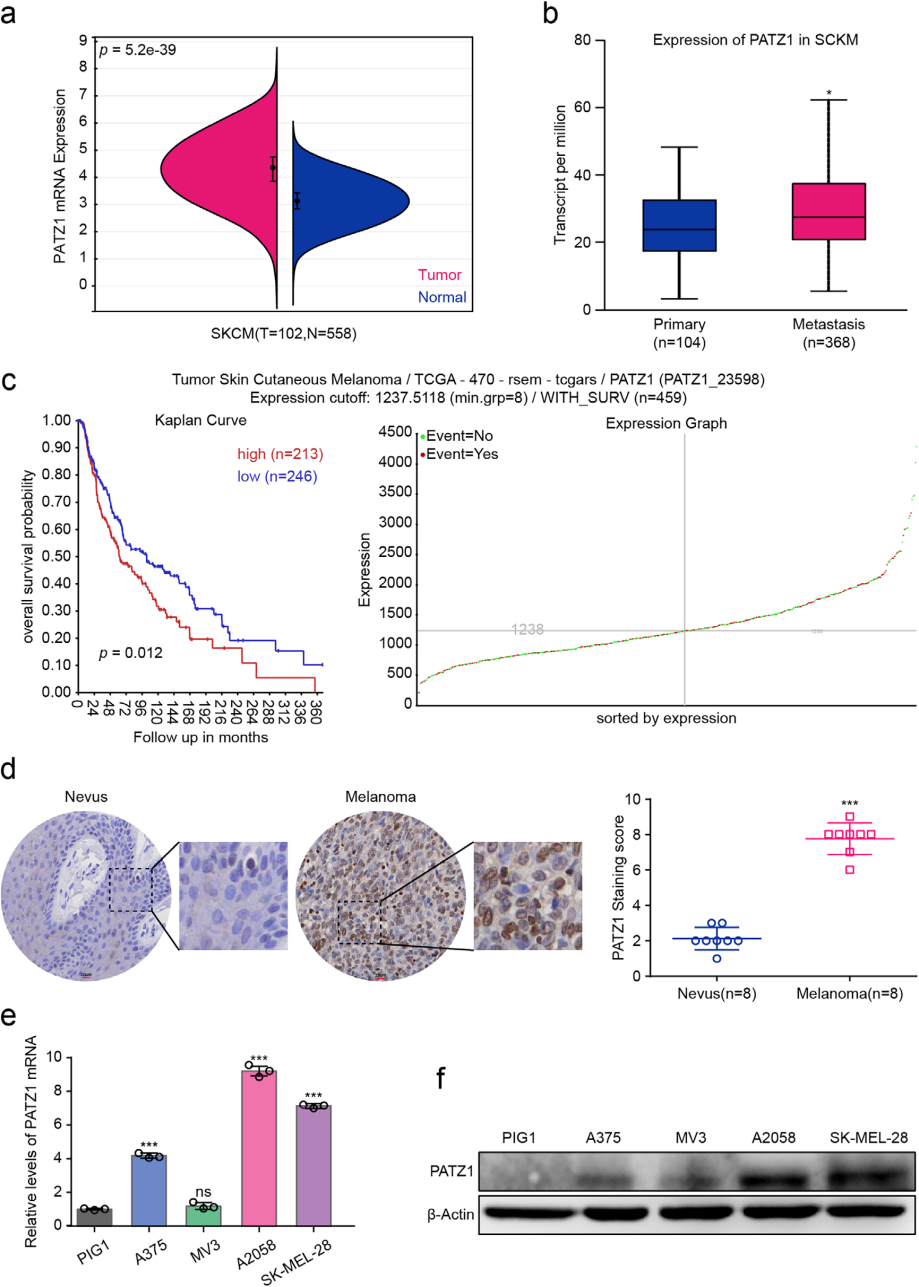
PATZ1 is overexpressed in melanoma and predicts poor patient survival. (a) PATZ1 mRNA expression in melanoma tissues versus normal skin tissues from the TCGA and GTEx cohorts (via Sangerbox). (b) PATZ1 mRNA expression in primary versus metastatic melanoma samples from the UALCAN database. (c) Kaplan–Meier overall survival (OS) analysis of melanoma patients stratified by high and low PATZ1 expression (TCGA data via R2). Patients were stratified into high‐ and low‐expression groups using the optimal cutoff determined by the R2 platform's log‐rank test scanning algorithm. (d) Representative immunohistochemistry (IHC) images showing PATZ1 protein expression in paired benign nevi and melanoma tissues (*n* = 8). (e) PATZ1 mRNA levels in a panel of melanoma cell lines compared to normal human melanocytes (PIG1). GAPDH was used as an internal reference. (f) PATZ1 protein levels in a panel of melanoma cell lines compared to normal human melanocytes (PIG1). β‐Actin was used as an internal reference. Each experiment was performed in triplicate. According to the data characteristics, quantitative data of (a,b,d,e) were analyzed by Student's *t*‐test, ^*^ *p* < 0.05, ^**^ *p* < 0.01, ^***^ *p* < 0.001.

To investigate the potential role of PATZ1 in melanoma, we first assessed its expression pattern and clinical relevance. Interrogation of public transcriptomic data from the TCGA and GTEx cohorts via the Sangerbox platform revealed that PATZ1 mRNA levels were significantly elevated in melanoma tissues compared to normal skin tissues (Figure 1a). Furthermore, analysis of the UALCAN database indicated that PATZ1 expression was even higher in metastatic melanomas than in primary tumors, suggesting a potential link to disease progression (Figure 1b). Critically, survival analyses conducted using the R2 Genomics Platform and Sangerbox demonstrated that high PATZ1 expression was strongly associated with poor overall patient survival (Figure 1c), as well as worse disease‐specific survival (DSS) and progression‐free interval (PFI) (Figure S1a). To validate these findings at the protein level, we performed immunohistochemistry (IHC) on a cohort of clinical specimens and observed consistently stronger PATZ1 staining in melanoma tissues (*n* = 8) compared to paired benign nevi (Figure 1d). This pronounced overexpression of PATZ1 was further confirmed in a panel of melanoma cell lines relative to normal human melanocytes (PIG1) by both qRT‐PCR and Western blot analysis (Figure 1e,f). Based on these results, we selected A2058 and SK‐MEL‐28 cell lines, which exhibit high endogenous PATZ1 expression, for subsequent functional investigations into its oncogenic role.

### 3.2 PATZ1 Drives the Aggressive Phenotype of Melanoma Cells In Vitro

**FIGURE 2:**
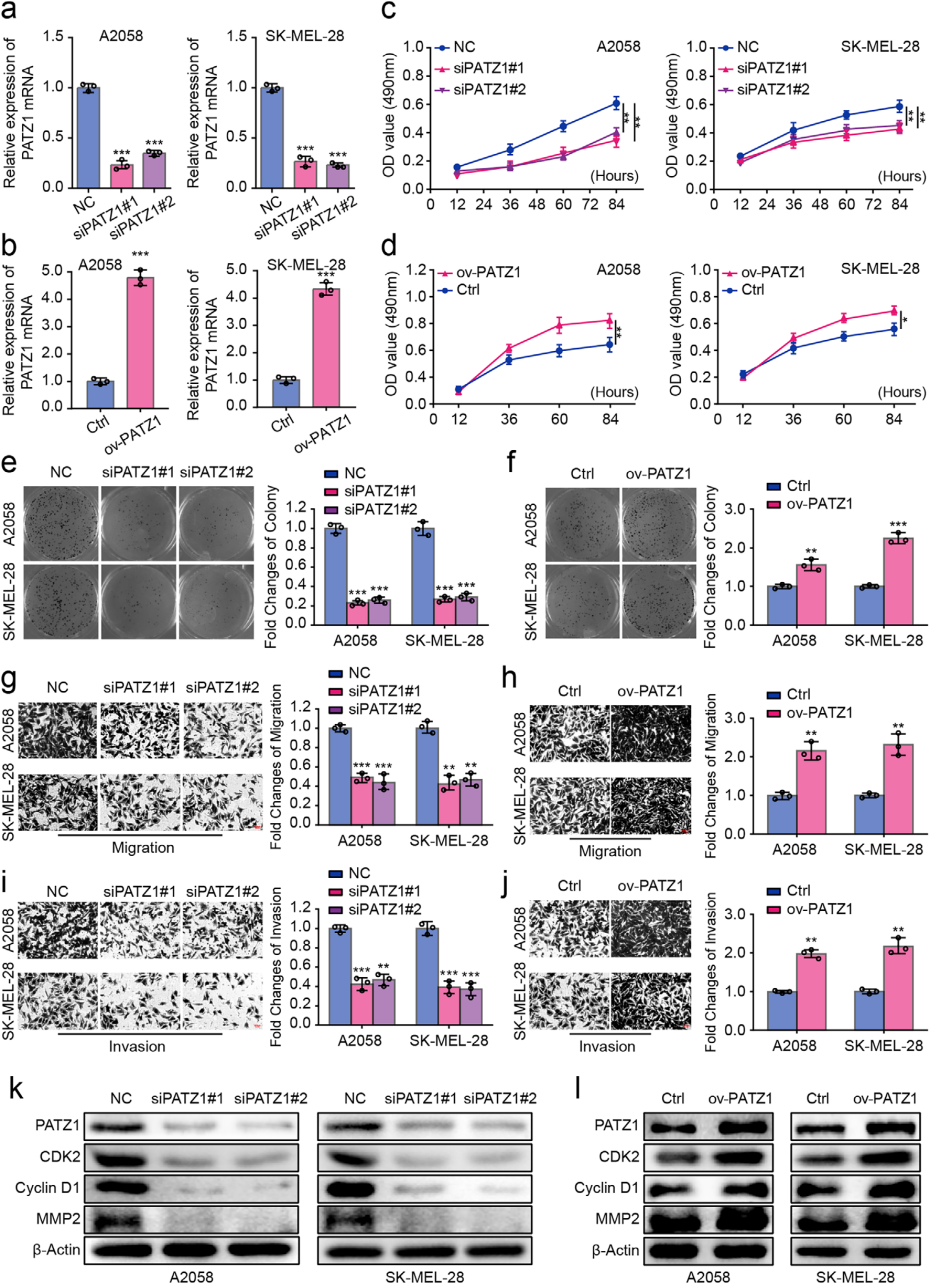
PATZ1 expression promotes melanoma cell proliferation, clonogenicity, migration, and invasion. (a,b) qRT‐PCR analysis confirming the efficiency of PATZ1 knockdown (a) and overexpression (b) in A2058 and SK‐MEL‐28 cells. GAPDH was used as an internal reference. (c,d) Cell viability assessed by MTT assay upon PATZ1 knockdown (c) and overexpression (d). (e,f) Clonogenic ability of melanoma cells upon PATZ1 knockdown (e) and overexpression (f). (g–j) Migratory (g,h) and invasive (i,j) capacities of melanoma cells evaluated by Transwell assays upon PATZ1 knockdown (g,i) and overexpression (h,j). (k,l) Western blot analysis of CDK2, Cyclin D1, and MMP2 protein levels upon PATZ1 knockdown (k) and overexpression (l). β‐Actin was used as an internal reference. Each experiment was performed in triplicate. According to the data characteristics, quantitative data of (a,b, e–j) were analyzed by Student's *t*‐test, quantitative data of (c,d) were analyzed by one‐way ANOVA followed by Tukey's post‐hoc test for multiple comparisons, ^*^ *p* < 0.05, ^**^ *p* < 0.01, ^***^ *p* < 0.001.

To investigate the functional role of PATZ1, we performed gain‐ and loss‐of‐function studies in A2058 and SK‐MEL‐28 melanoma cells, with efficient manipulation confirmed by qRT‐PCR (Figure 2a,b). MTT assays demonstrated that PATZ1 knockdown significantly reduced cell viability, while its overexpression enhanced it (Figure 2c,d). This pro‐proliferative role was further supported by clonogenic assays, which showed that PATZ1 depletion markedly impaired long‐term colony‐forming capacity, an effect that was augmented upon PATZ1 overexpression (Figure 2e,f). Furthermore, Transwell migration and invasion assays revealed that PATZ1 knockdown potently suppressed, while its overexpression significantly enhanced, the migratory and invasive capabilities of melanoma cells (Figure 2g–j). Analysis of cell fate indicated that PATZ1 knockdown induced profound apoptosis and cell cycle arrest at the G0/G1 phase, whereas its overexpression suppressed apoptosis and promoted cell cycle progression (Figure S2a–h). The regulatory role of PATZ1 was further elucidated at the molecular level by Western blot, which demonstrated that PATZ1 knockdown downregulated the cell cycle promoters CDK2 and cyclin D1, and the metastasis‐associated protein MMP2, whereas PATZ1 overexpression had the opposite effects (Figure 2k,l).

To examine whether the oncogenic function of PATZ1 extends beyond the initially characterized cell models, we extended our analysis to the SK‐MEL‐2 cell line, which harbors a distinct NRAS Q61R mutation. Efficient knockdown of PATZ1 in SK‐MEL‐2 cells was first confirmed (Figure S3a,b). Consistent with our findings in A2058 and SK‐MEL‐28 cells, depletion of PATZ1 in this NRAS‐mutant context significantly impaired cell viability, clonogenic potential, as well as migratory and invasive capabilities (Figure S3c–g). These results reinforce that the tumor‐promoting role of PATZ1 is not restricted to a specific genetic background but is operative across melanoma subtypes driven by major oncogenic mutations.

Collectively, these in vitro findings establish PATZ1 as a key driver of melanoma cell proliferation, clonogenicity, and invasion, thereby justifying further investigation into its tumorigenic capacity in vivo.

### 3.3 PATZ1 Knockdown Suppresses Melanoma Tumor Growth In Vivo

**FIGURE 3:**
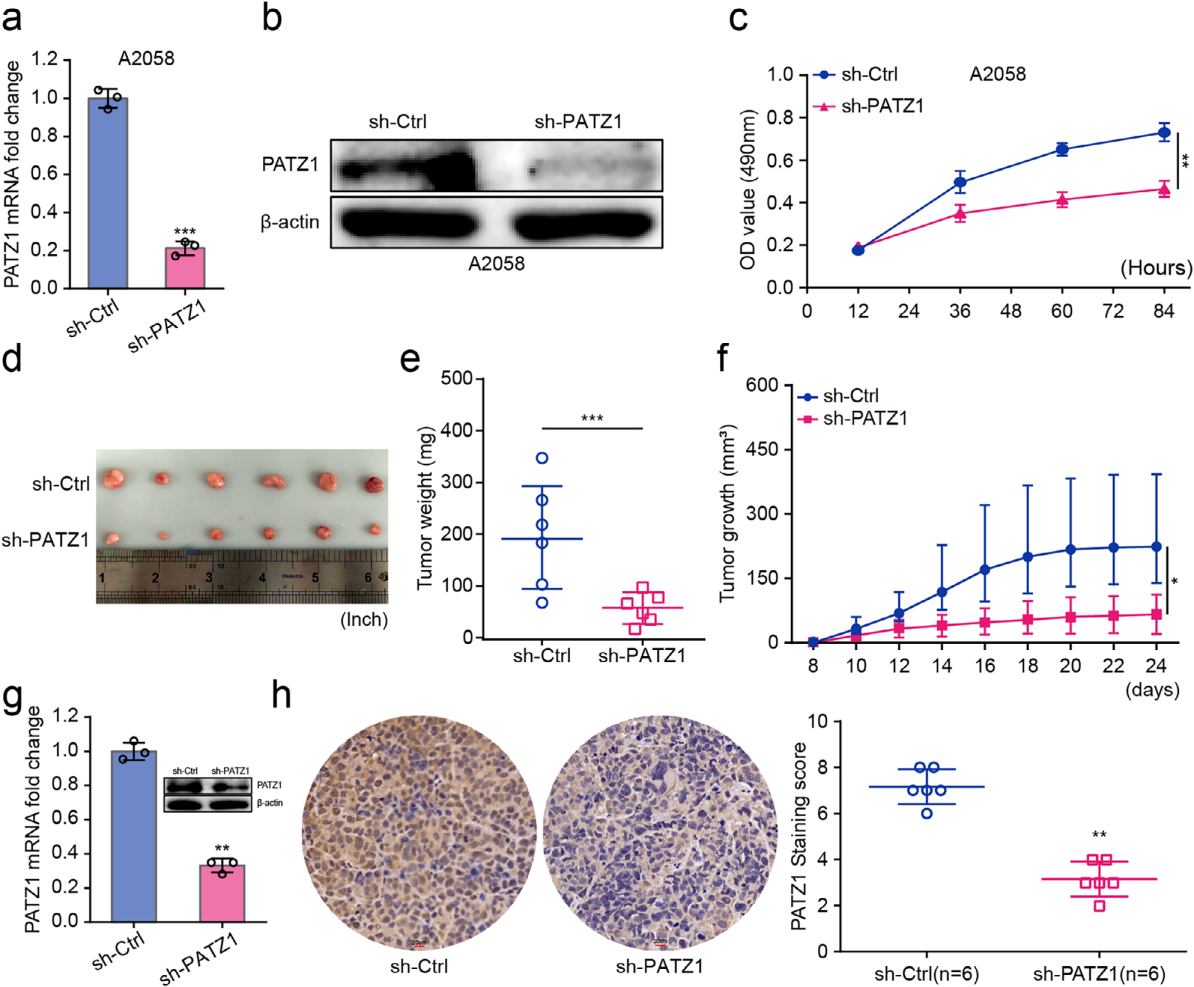
PATZ1 knockdown suppresses melanoma tumor growth in vivo. (a) Validation of stable PATZ1 knockdown in A2058 cells by qRT‐PCR. GAPDH was used as an internal reference. (b) Validation of stable PATZ1 knockdown in A2058 cells by Western blot. β‐Actin was used as an internal reference. (c) MTT assay showing cell viability of stable PATZ1‐knockdown cells in vitro. (d–f) In vivo tumor growth in nude mice injected subcutaneously with control or PATZ1‐knockdown A2058 cells. Tumor images (d), growth curves (e), and weights (f) are shown (*n* = 6 mice per group). (g) Validation of PATZ1 knockdown in the resected xenograft tumors by qRT‐PCR (GAPDH was used as an internal reference), Western blot (β‐Actin was used as an internal reference). (h) Validation of PATZ1 knockdown in the resected xenograft tumors by IHC. Each experiment was performed in triplicate. According to the data characteristics, quantitative data of (a,e,g,h) were analyzed by Student's *t*‐test, quantitative data of (c,f) were analyzed by one‐way ANOVA followed by Tukey's post‐hoc test for multiple comparisons, ^*^ *p* < 0.05, ^**^ *p* < 0.01, ^***^ *p* < 0.001.

To substantiate our in vitro findings, we sought to determine whether PATZ1 is required for melanoma tumor growth in vivo. We established a stable PATZ1‐knockdown A2058 cell line using a shRNA lentivirus transfection system, which resulted in a significant decrease in both PATZ1 mRNA and protein levels, as confirmed by qRT‐PCR and Western blot analysis (Figure 3a,b). The diminished oncogenic capacity of these cells was first verified in vitro by MTT assay, which recapitulated the reduction in cell viability observed with transient knockdown (Figure 3c). These cells were then subcutaneously injected into nude mice to monitor tumor formation. Consistent with its tumor‐promoting role, PATZ1 knockdown profoundly impaired the tumorigenic potential of melanoma cells in vivo, leading to a significant reduction in both tumor volume and weight compared to the control group (Figure 3d–f). The successful downregulation of PATZ1 in the resultant xenograft tumors was further validated by qRT‐PCR, Western blot, and immunohistochemistry (Figure 3g,h). Having firmly established PATZ1 as a bona fide oncogenic driver both in vitro and in vivo, we next aimed to elucidate the underlying molecular mechanism by which it exerts its pro‐tumorigenic effects.

### 3.4 PATZ1 and CTCF Antagonistically Regulate the Tumor Suppressor ZBTB20

**FIGURE 4:**
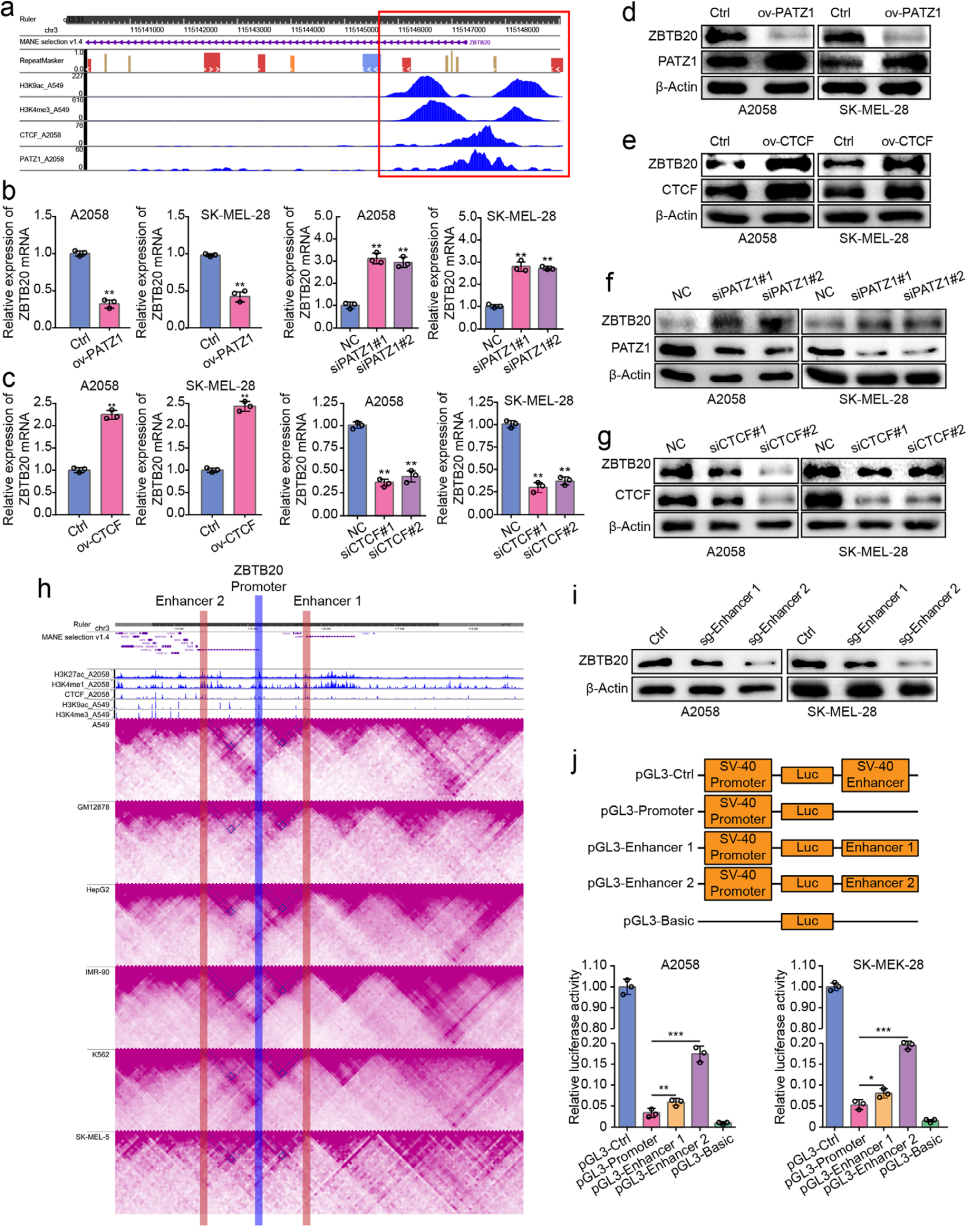
PATZ1 and CTCF antagonistically regulate the tumor suppressor ZBTB20. (a) ZBTB20 was identified as a top candidate target from the intersection, visualized via the WashU Epigenome Browser. (b) ZBTB20 mRNA levels upon PATZ1 Knockdown or overexpression. GAPDH was used as an internal reference. (c) ZBTB20 mRNA levels upon CTCF Knockdown or overexpression. GAPDH was used as an internal reference. (d) ZBTB20 protein levels upon PATZ1 overexpression. β‐Actin was used as an internal reference. (e) ZBTB20 protein levels upon CTCF overexpression. β‐Actin was used as an internal reference. (f) ZBTB20 protein levels upon PATZ1 Knockdown. β‐Actin was used as an internal reference. (g) ZBTB20 protein levels upon CTCF Knockdown. β‐Actin was used as an internal reference. (g) ZBTB20 protein levels upon CTCF overexpression. β‐Actin was used as an internal reference. (h) Hi‐C interaction map and chromatin state (H3K4me1/H3K27ac CUT&Tag) at the ZBTB20 locus, identifying two candidate enhancers (Enhancer1, Enhancer2) within the same TAD. (i) ZBTB20 mRNA levels after CRISPR/Cas9‐mediated deletion of Enhancer1 or Enhancer2. β‐Actin was used as an internal reference. (j) Dual‐luciferase reporter assay assessing the transcriptional activity of the ZBTB20 promoter when coupled with Enhancer1 or Enhancer2. Each experiment was performed in triplicate. According to the data characteristics, quantitative data of (b,c,j) were analyzed by Student's *t*‐test, ^*^ *p* < 0.05, ^**^ *p* < 0.01, ^***^ *p* < 0.001.

To elucidate the oncogenic mechanism of PATZ1, we first investigated its DNA‐binding properties. Bioinformatics analysis revealed that the DNA‐binding motif of PATZ1 shares a high degree of similarity with that of the architectural protein CTCF, and their chromatin binding sites exhibit significant genome‐wide co‐localization (Figure S4a,b), suggesting potential functional interplay. However, co‐immunoprecipitation assays ruled out the formation of a stable protein–protein complex between PATZ1 and CTCF (Figure S4c), directing our focus to a novel model of competitive DNA binding. Given CTCF's canonical role in orchestrating enhancer‐promoter interactions via chromatin looping, we hypothesized that PATZ1 might drive tumorigenesis by mimicking and competitively disrupting CTCF‐mediated chromatin architecture. To test this, we integrated PATZ1 and CTCF CUT&Tag data with transcriptomic profiles to identify genes repressed by PATZ1 yet activated by CTCF. This approach pinpointed ZBTB20, a gene whose expression showed a significant negative correlation with PATZ1 and a positive correlation with CTCF in melanoma cohorts (Figure 4a; Figure S4d,e). Functional validation confirmed that PATZ1 overexpression suppressed while its knockdown elevated ZBTB20 expression; conversely, CTCF overexpression enhanced while its knockdown reduced ZBTB20 levels (Figure 4b–g). A dual‐luciferase reporter assay demonstrated that PATZ1 overexpression suppressed ZBTB20 promoter activity, whereas CTCF overexpression did not enhance it (Figure S4f), suggesting that CTCF's regulatory role might depend on higher‐order chromatin context not recapitulated in this assay. Supporting this notion, ZBTB20 expression positively correlated with cohesin subunits (SMC1, RAD21) (Figure S4g), and public ChIP‐seq data confirmed the binding of these subunits to the ZBTB20 promoter (Figure S4h), strongly implying the existence of a CTCF/cohesin‐mediated chromatin loop at this locus. To define this regulatory architecture, we first employed EnhancerAtlas to predict potential enhancers regulating ZBTB20 (Figure S4i) and subsequently integrated H3K4me1/H3K27ac CUT&Tag signals with public Hi‐C data, which identified two specific enhancers located within the same topologically associating domain as the ZBTB20 promoter (Figure 4h). CRISPR/Cas9‐mediated deletion of either enhancer significantly reduced ZBTB20 expression (Figure 4i; Figure S4j), and dual‐luciferase assays confirmed that each enhancer could independently and robustly activate the promoter (Figure 4j). Collectively, this segment not only identifies ZBTB20 as a key target under antagonistic control by PATZ1 and CTCF but, critically, establishes that ZBTB20 transcription is governed by a specific chromatin loop structure, thereby setting the mechanistic stage for investigating how PATZ1 disrupts this architecture to silence tumor suppression.

### 3.5 PATZ1 Competitively Disrupts a CTCF‐Dependent Chromatin Loop to Silence ZBTB20

**FIGURE 5:**
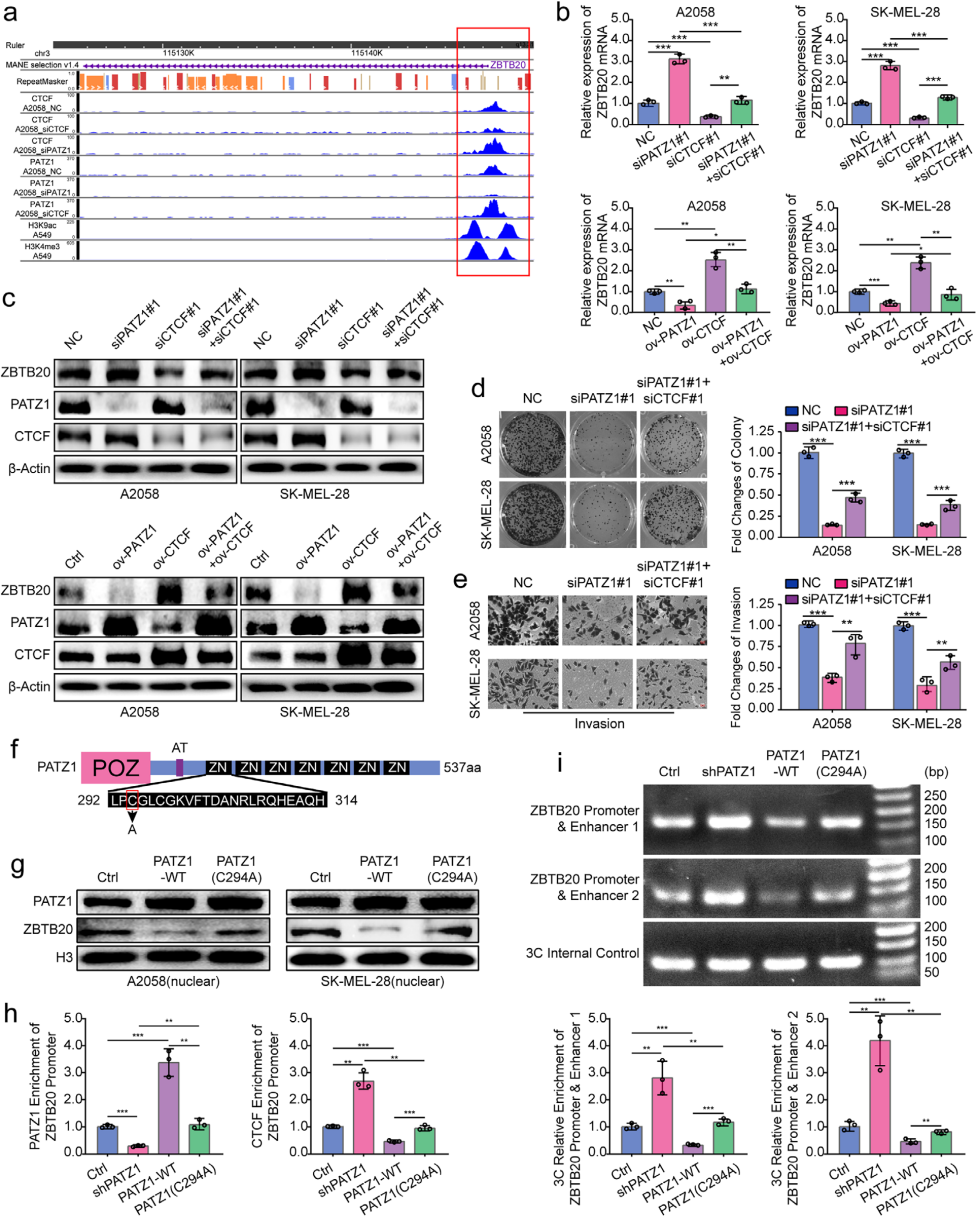
PATZ1 competitively disrupts a CTCF‐dependent chromatin loop to silence ZBTB20. (a) WashU Epigenome Browser snapshot of the ZBTB20 promoter region. Tracks display CUT&Tag sequencing signals for PATZ1 and CTCF in control (siCtrl), PATZ1‐knockdown (siPATZ1), and CTCF‐knockdown (siCTCF) A2058 cells, demonstrating reciprocal binding changes. (b) qPCR showing ZBTB20 mRNA levels under single or combined manipulations of PATZ1 and CTCF. GAPDH was used as an internal reference. (c) Western blot showing ZBTB20 protein levels under single or combined manipulations of PATZ1 and CTCF. β‐Actin was used as an internal reference. (d) Colony formation assays in A2058 cells transfected with the indicated siRNAs. (e) Transwell invasion assays in A2058 cells transfected with the indicated siRNAs. (f) Schematic diagram of PATZ1 DNA binding mutant (C294A). (g) Western blot analysis of PATZ1 and ZBTB20 expression levels in nuclear extracts protein of wild‐type PATZ1 (PATZ1‐WT) and the PATZ1(C294A) mutant in transfected A2058 cells. H3 was used as an internal reference. (h) Quantitative CUT&Tag‐qPCR analysis of PATZ1 and CTCF occupancy at the endogenous ZBTB20 promoter in A2058 cells expressing control shRNA (shCtrl), PATZ1‐targeting shRNA (shPATZ1), PATZ1‐WT, or PATZ1(C294A). (i) Chromosome conformation capture (3C) analysis quantifying the interaction frequency between the ZBTB20 promoter and Enhancer 1/Enhancer 2 in A2058 cells stably expressing control shRNA (shCtrl), PATZ1‐targeting shRNA (shPATZ1), PATZ1‐WT, or PATZ1(C294A). Interaction frequency is normalized to a control genomic region and expressed relative to shCtrl. Each experiment was performed in triplicate. According to the data characteristics, quantitative data of (b,d,h,i) were analyzed by Student's *t*‐test, ^*^ *p* < 0.05, ^**^ *p* < 0.01, ^***^ *p* < 0.001.

Having established ZBTB20 as a target antagonistically regulated by PATZ1 and CTCF via a chromatin loop, we next directly validated the proposed competition model and its consequences. Interrogation of our genome‐wide CUT&Tag sequencing data via the WashU Epigenome Browser revealed that knockdown of PATZ1 markedly enhanced the CTCF binding signal at the ZBTB20 promoter, while knockdown of CTCF increased the PATZ1 signal at this shared site, providing direct visual evidence of a reciprocal competitive relationship in vivo (Figure 5a). Functionally, this competition governed ZBTB20 expression and oncogenic phenotypes: PATZ1 effectively antagonized CTCF‐mediated transcriptional activation of ZBTB20 (Figure 5b,c), and concomitant knockdown of CTCF significantly rescued the suppression of clonogenicity and invasion caused by PATZ1 depletion alone (Figure 5d,e). To definitively prove that this competition requires PATZ1's sequence‐specific DNA‐binding ability, we employed a DNA‐binding‐deficient point mutant (PATZ1‐C294A). While expressed and localized normally (Figure 5f,g; Figure S5a), this mutant was transcriptionally inert, as it failed to repress the ZBTB20 promoter in a dual‐luciferase reporter assay (Figure S5b). Accordingly, and in contrast to wild‐type PATZ1, the PATZ1‐C294A mutant could not alter the occupancy of either PATZ1 or CTCF at the endogenous ZBTB20 locus as quantified by CUT&Tag‐qPCR (Figure 5h). The ultimate structural consequence of this DNA‐binding‐dependent competition was the dissolution of the chromatin loop itself. Chromosome conformation capture (3C) assays demonstrated that PATZ1 knockdown enhanced, while PATZ1‐WT overexpression weakened, the promoter‐enhancer interaction; critically, the PATZ1‐C294A mutant had no effect on the loop strength (Figure 5i). Thus, we provide a coherent causal chain wherein PATZ1, via direct competition with CTCF for promoter occupancy, disrupts the specific chromatin loop architecture to silence ZBTB20. This positions the epigenetic inactivation of ZBTB20 as the central mechanistic link driving oncogenic reprogramming, leading us to define its tumor‐suppressive functions.

### 3.6 ZBTB20 Functions as a Tumor Suppressor in Melanoma

The ZBTB family of transcription factors, characterized by their N‐terminal BTB domain for protein interaction and C‐terminal zinc finger domains for DNA binding, are evolutionarily conserved regulators of development, differentiation, and immunity [53]. Several members, including the tumor suppressor ZBTB16 and the oncogene ZBTB7A, are well‐established players in cancer pathogenesis [54, 55]. Among them, ZBTB20 has emerged as a significant yet context‐dependent regulator in multiple malignancies [56]. It functions as an oncogene in hepatocellular carcinoma (HCC) by repressing FOXO1, in gastric adenocarcinoma by activating NF‐κB, and promotes progression in non‐small cell lung cancer, glioblastoma, breast cancer, and acute myeloid leukemia [57, 58, 59, 60, 61, 62]. Conversely, contrasting reports also imply potential tumor‐suppressive roles: in HCC, its repression by specific regulatory axes is linked to aggressive tumor behavior, and its depletion in hepatocytes enhances viral infection associated with carcinogenesis [63]. Furthermore, in the tumor immune microenvironment, deletion of ZBTB20 in CD8+ T cells enhances their anti‐tumor activity, suggesting a cell‐type‐specific role in restraining immunity [64]. Despite this extensive and complex involvement across cancer types, the biological function and clinical significance of ZBTB20 in melanoma have remained entirely unexplored. Given our discovery that ZBTB20 is a key transcriptional target silenced by the PATZ1/CTCF balance, we proceeded to systematically investigate its functional impact in this malignancy.

**FIGURE 6:**
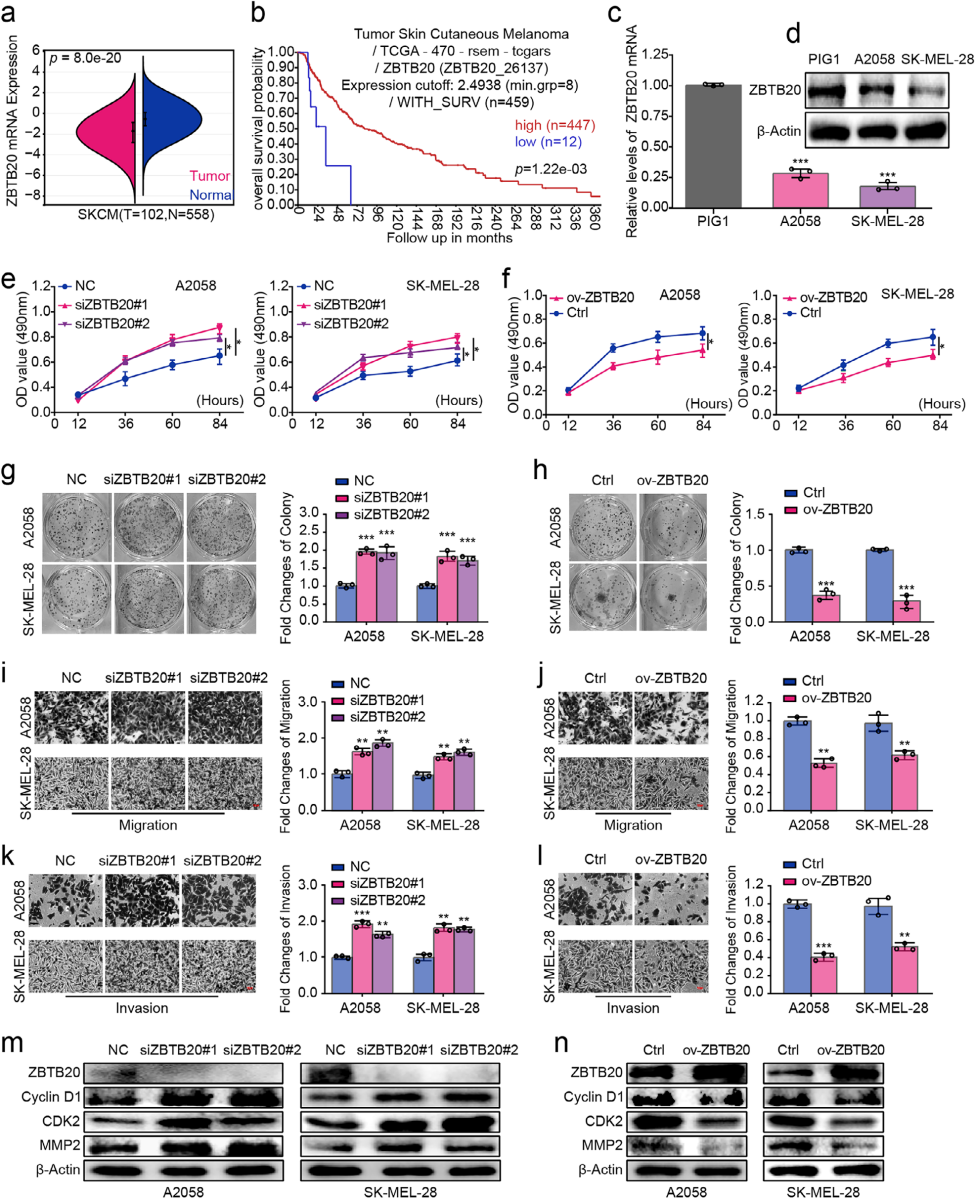
ZBTB20 functions as a tumor suppressor in melanoma. (a) ZBTB20 mRNA expression in melanoma versus normal skin from TCGA/GTEx (via Sangerbox). (b) Kaplan–Meier OS analysis of melanoma patients stratified by ZBTB20 expression. Patients were stratified into high‐ and low‐expression groups using the optimal cutoff determined by the R2 platform's log‐rank test scanning algorithm. (c) ZBTB20 mRNA levels in melanoma cell lines versus PIG1 cells. GAPDH was used as an internal reference. (d) ZBTB20 protein levels in melanoma cell lines versus PIG1 cells. β‐Actin was used as an internal reference. (e–h) Cell viability (e,f; MTT) and clonogenicity (g,h) upon ZBTB20 knockdown (e,g) and overexpression (f,h). (i–l) Migratory (i,j) and invasive (k,l) capacities upon ZBTB20 knockdown (i,k) and overexpression (j,l). (m,n) Western blot analysis of CDK2, Cyclin D1, and MMP2 upon ZBTB20 knockdown (m) and overexpression (n). β‐Actin was used as an internal reference. Each experiment was performed in triplicate. According to the data characteristics, quantitative data of (a,c, g–l) were analyzed by Student's *t*‐test, quantitative data of (e,f) were analyzed by one‐way ANOVA followed by Tukey's post‐hoc test for multiple comparisons, ^*^ *p* < 0.05, ^**^ *p* < 0.01, ^***^ *p* < 0.001.

To this end, we first assessed the clinical relevance of ZBTB20 in melanoma. Interrogation of the TCGA and GTEx cohorts via Sangerbox revealed that ZBTB20 mRNA levels were significantly lower in melanoma tissues compared to normal skin (Figure 6a). Survival analyses conducted using the R2 platform and Sangerbox demonstrated that low ZBTB20 expression was strongly associated with poor overall survival (Figure 6b), as well as worse disease‐specific survival (DSS) and progression‐free interval (PFI) (Figure S5a). Furthermore, analysis of the UALCAN database indicated that ZBTB20 expression correlated with clinical stages and nodal metastasis status (Figure S5b). This reduced expression of ZBTB20 in melanoma cell lines (A2058 and SK‐MEL‐28) relative to normal human melanocytes was confirmed at both the mRNA and protein levels by qRT‐PCR and Western blot (Figure 6c,d). To determine the functional consequences of ZBTB20 loss, we performed gain‐ and loss‐of‐function studies. Efficient knockdown and overexpression of ZBTB20 were confirmed by qRT‐PCR (Figure S5c,d). MTT and clonogenic assays showed that ZBTB20 depletion enhanced cell viability and colony formation, whereas its overexpression suppressed these phenotypes (Figure 6e–h). Similarly, ZBTB20 knockdown potentiated, while its overexpression inhibited, melanoma cell migration and invasion in Transwell assays (Figure 6i–l). Cell cycle analysis by flow cytometry revealed that ZBTB20 knockdown promoted G1/S phase transition, whereas its overexpression induced G0/G1 arrest (Figure S5e–h). Consistent with these phenotypic changes, Western blot analysis demonstrated that ZBTB20 knockdown upregulated key cell cycle promoters (CDK2, cyclin D1) and the metastasis‐associated protein MMP2, which were downregulated upon ZBTB20 overexpression (Figure 6m,n). Collectively, these findings establish ZBTB20 as a potent tumor suppressor in melanoma, whose loss promotes aggressive tumorigenic behaviors. This positioned ZBTB20 as the critical downstream effector whose silencing is central to the oncogenic phenotype driven by PATZ1.

### 3.7 ZBTB20 is Essential for PATZ1‐Mediated Oncogenicity

**FIGURE 7:**
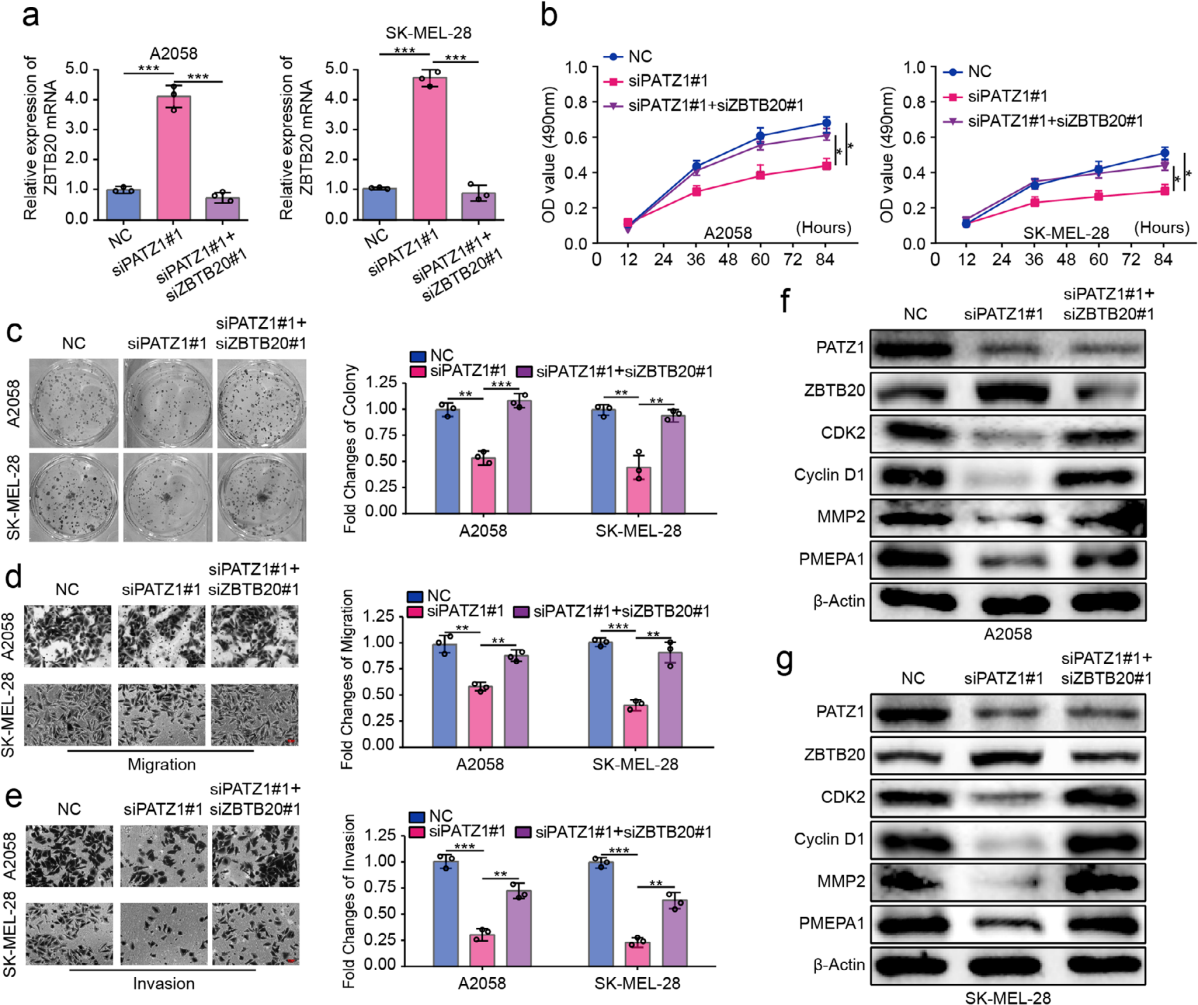
ZBTB20 is essential for PATZ1‐mediated oncogenicity. (a) qRT‐PCR confirming ZBTB20 expression in rescue experiments. GAPDH was used as an internal reference. (b–e) Functional rescue assays: MTT (b), clonogenic (c), migration (d), and invasion (e) assays in cells with PATZ1 knockdown alone or combined with ZBTB20 knockdown. (f,g) Western blot analysis of CDK2, Cyclin D1, and MMP2 in the rescue setting. β‐Actin was used as an internal reference. Each experiment was performed in triplicate. According to the data characteristics, quantitative data of (a, c–e) were analyzed by Student's *t*‐test, quantitative data of (b) were analyzed by one‐way ANOVA followed by Tukey's post‐hoc test for multiple comparisons, ^*^ *p* < 0.05, ^**^ *p* < 0.01, ^***^ *p* < 0.001.

Our data have so far demonstrated that PATZ1 represses the expression of the tumor suppressor ZBTB20 and that loss of ZBTB20 promotes oncogenic phenotypes. We next sought to determine whether the repression of ZBTB20 is functionally indispensable for PATZ1‐driven tumor progression. To test this, we performed a series of genetic rescue experiments in A2058 and SK‐MEL‐28 cells. We confirmed by qRT‐PCR that single knockdown of PATZ1 successfully upregulated ZBTB20 expression, while the concomitant knockdown of ZBTB20 in this setting effectively restored ZBTB20 to near‐baseline levels (Figure 7a). Functionally, PATZ1 knockdown significantly suppressed cell viability in MTT assays, impaired clonogenic capacity, and reduced cell migration and invasion in Transwell assays (Figure 7b–e). Strikingly, co‐knockdown of ZBTB20 largely rescued these tumor‐suppressive effects, restoring cell proliferation, colony formation, and metastatic potential to levels comparable to the control (Figure 7b–e). This rescuing effect was further corroborated at the molecular level by Western blot analysis, which showed that the downregulation of CDK2, cyclin D1, and MMP2 induced by PATZ1 knockdown was reversed upon simultaneous ZBTB20 depletion (Figure 7f,g). Collectively, these data unequivocally demonstrate that the transcriptional repression of ZBTB20 is a critical downstream event through which PATZ1 exerts its oncogenic functions, cementing ZBTB20's role as the essential effector in this pathogenic axis.

### 3.8 ZBTB20 Exerts Its Tumor‐Suppressive Function by Repressing the PMEPA1‐p38‐STAT1 Oncogenic Signaling Axis

**FIGURE 8:**
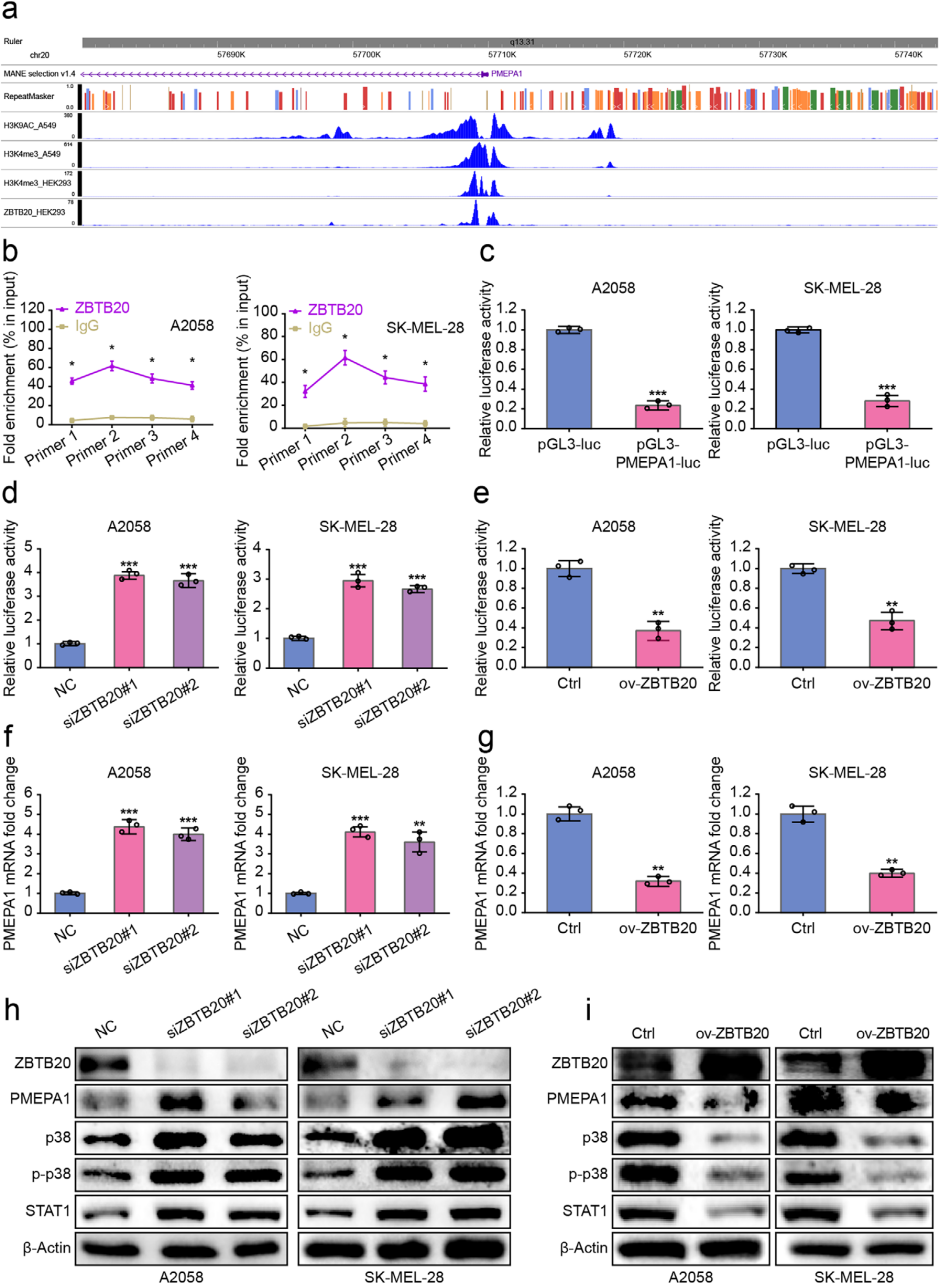
ZBTB20 exerts its tumor‐suppressive function by repressing the PMEPA1‐p38‐STAT1 oncogenic signaling axis. (a) Integration of ZBTB20 ChIP‐seq and RNA‐seq data nominating PMEPA1 as a direct target. (b) CUT&Tag‐qPCR confirming ZBTB20 binding to the PMEPA1 promoter. (c,d) Dual‐luciferase reporter assay of PMEPA1 promoter activity upon ZBTB20 knockdown (c) and overexpression (d). (f,g) PMEPA1 mRNA levels upon ZBTB20 knockdown (f) and overexpression (g). GAPDH was used as an internal reference. (h,i) PMEPA1 protein levels, and corresponding levels of p38, p‐p38, and STAT1, upon ZBTB20 knockdown (h) and overexpression (i). β‐Actin was used as an internal reference. Each experiment was performed in triplicate. According to the data characteristics, quantitative data of (c–g) were analyzed by Student's *t*‐test, quantitative data of (b) were analyzed by one‐way ANOVA followed by Tukey's post‐hoc test for multiple comparisons, ^*^ *p* < 0.05, ^**^ *p* < 0.01, ^***^ *p* < 0.001.

Having established ZBTB20 as the essential downstream effector of PATZ1, we next sought to delineate the mechanism by which ZBTB20 itself constrains melanoma progression. To identify direct transcriptional targets of ZBTB20, we integrated data from public ZBTB20 ChIP‐seq datasets with our own transcriptomic profiling upon ZBTB20 manipulation. This analysis nominated PMEPA1, a known negative feedback regulator of TGF‐β signaling reported to activate non‐canonical pathways like MAPK [65], as a prime candidate (Figure 8a). We confirmed that ZBTB20 directly binds to the promoter region of PMEPA1 by CUT&Tag‐qPCR (Figure 8b). Analysis of publicly available melanoma cohorts via cBioPortal revealed a significant negative correlation between ZBTB20 and PMEPA1 mRNA expression (Figure S6a), providing compelling clinical evidence for this regulatory relationship. Consistent with its role as a transcriptional repressor, dual‐luciferase reporter assays demonstrated that ZBTB20 knockdown enhanced, while its overexpression suppressed, PMEPA1 promoter activity (Figure 8c,d). Accordingly, manipulations of ZBTB20 levels produced inverse changes in PMEPA1 mRNA and protein expression, whereby ZBTB20 knockdown upregulated, and overexpression downregulated PMEPA1 (Figure 8f–i).

Given the documented role of PMEPA1 in activating pro‐tumorigenic non‐canonical signaling, we interrogated its downstream pathway. Western blot analysis revealed that knockdown of ZBTB20, which led to PMEPA1 upregulation, resulted in a marked increase in the phosphorylation of p38, consistent with pathway activation. Concomitantly, we observed an increase in the protein level of the key downstream effector STAT1, indicating hyperactivation of the p38‐STAT1 signaling axis (Figure 8h). Overexpression of ZBTB20 produced the opposite effect (Figure 8i).

**FIGURE 9:**
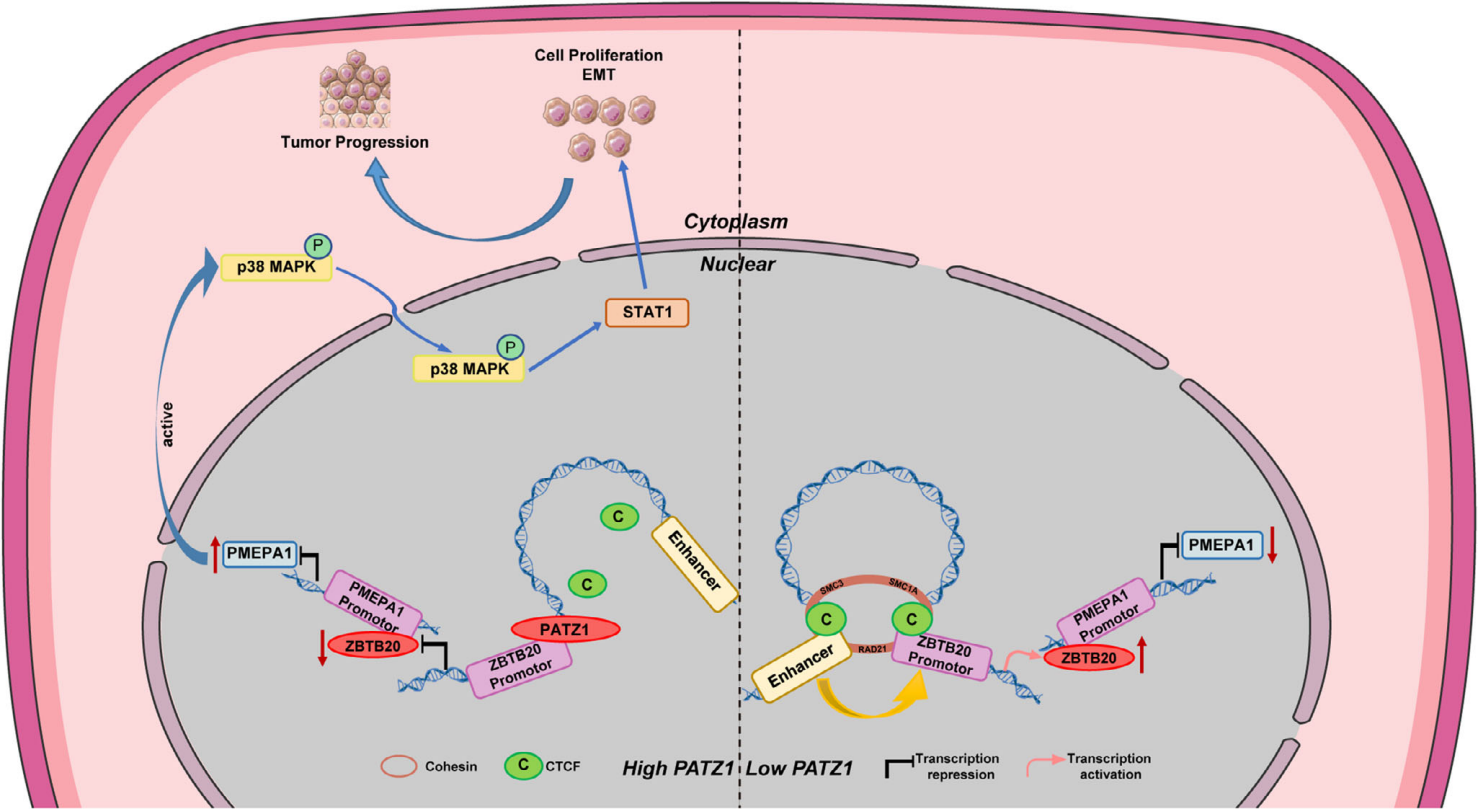
A working model of the PATZ1/CTCF‐ZBTB20‐PMEPA1‐p38‐STAT1 oncogenic axis. In normal melanocytes, CTCF binding facilitates the formation of a chromatin loop between enhancers and the ZBTB20 promoter, maintaining ZBTB20 expression, which represses PMEPA1 and inhibits p38‐STAT1 signaling. In melanoma, aberrant PATZ1 overexpression competitively displaces CTCF, leading to the collapse of the chromatin loop, epigenetic silencing of ZBTB20, and consequent derepression of PMEPA1. Elevated PMEPA1 activates the p38‐STAT1 signaling pathway, ultimately driving melanoma progression.

Collectively, our findings unveil a previously unrecognized oncogenic pathway in melanoma, orchestrated by the disruption of a dynamic balance between PATZ1 and CTCF. In normal physiology, CTCF predominance at the ZBTB20 locus maintains an active chromatin state permissive for transcription. In melanoma, however, this homeostatic balance is skewed by aberrant PATZ1 overexpression. PATZ1 competes with and displaces CTCF, leading to the collapse of the enhancer‐promoter loop and epigenetic silencing of the tumor suppressor ZBTB20. The downregulation of ZBTB20, in turn, de‐represses the proto‐oncogene PMEPA1, whose elevated expression drives the activation of the pro‐tumorigenic p38‐STAT1 signaling pathway, ultimately fueling melanoma progression (Figure 9). This work elucidates a complete pathogenic axis—from the dysregulation of a transcriptional balance to aberrant signal transduction—the “PATZ1/CTCF‐ZBTB20‐PMEPA1‐p38‐STAT1” axis.

### 3.9 Exploratory Analysis of Potential Upstream Drivers of PATZ1 Overexpression

To gain initial insights into the mechanisms underlying PATZ1 overexpression in melanoma—a premise central to our study—we conducted a series of bioinformatic analyses using publicly available melanoma genomics datasets.

Interrogation of the TCGA‐SKCM cohort via cBioPortal revealed that the PATZ1 locus undergoes copy‐number gain or amplification in a definable subset of melanoma samples (Figure S8a). Consistent with a potential gene‐dosage effect, PATZ1 copy‐number alteration showed a positive correlation with its mRNA expression level across the cohort (Figure S8b), suggesting genomic amplification as one contributory mechanism. In contrast, analysis of DNA methylation at the PATZ1 promoter region did not reveal a significant inverse correlation with its expression (Figure S8c), indicating that promoter hypomethylation is unlikely a primary driver.

To identify potential transcriptional regulators, we integrated predictions from multiple databases. Interestingly, while the oncogenic transcription factor STAT3 was predicted to bind the PATZ1 promoter, its expression correlated negatively with PATZ1 mRNA levels in patient samples (Figure S8d,e), hinting at a complex or context‐dependent regulatory relationship. Most notably, we discovered a strong positive correlation between the mRNA levels of PATZ1 and MITF, the master lineage transcription factor in melanoma (Figure S8f). This compelling association suggests that PATZ1 may be integrated into the MITF‐driven transcriptional program, providing a plausible explanation for its cell type‐specific overexpression. These exploratory analyses nominate genomic copy‐number alteration and a potential link to the melanocyte lineage program as upstream mechanisms worthy of further experimental investigation. These bioinformatic associations suggest potential mechanisms underlying PATZ1 dysregulation, the implications of which we explore in the context of our functional findings below.

## 4 Discussion

In this study, we delineate a novel oncogenic axis in melanoma, wherein the aberrant overexpression of PATZ1 drives tumor progression by competitively disrupting CTCF‐mediated chromatin architecture to silence the tumor suppressor ZBTB20, thereby unleashing the PMEPA1‐p38‐STAT1 signaling cascade. Our work positions PATZ1 as a pivotal oncogenic driver and, more importantly, unveils a new paradigm of epigenetic dysregulation centered on the disruption of transcriptional balance at the chromatin level.

The most salient finding of our research is the proposal and rigorous validation of the “PATZ1/CTCF dynamic balance” as a fundamental regulatory mechanism. CTCF is a well‐established critical chromatin architectural protein and a master organizer of the 3D genome [66, 67, 68, 69, 70, 71, 72, 73, 74]. Its fundamental role is to orchestrate interactions between regulatory elements and help separate eu‐ and heterochromatic areas in the genome, exerting a chromatin barrier function [25]. This is achieved through the formation of topologically associating domains (TADs), which are intricate in their structures and frequently feature smaller domains referred to as sub‐TADs, thereby ensuring proper promoter‐enhancer dialogues [25]. Our work fundamentally extends the understanding of CTCF dysregulation in cancer. The prevailing paradigm attributes such dysregulation primarily to genetic alterations in CTCF itself or its binding sites, or to changes in its expression level. Here, we describe a conceptually distinct mechanism: the functional hijacking of CTCF‐mediated chromatin organization by a competing transcription factor, PATZ1, whose overexpression tips the balance at specific loci without necessarily altering CTCF's genomic presence or sequence specificity. Our data demonstrate that CTCF's function can be subverted through direct, DNA‐binding‐dependent competition for motif occupancy. The structural similarity between PATZ1 and CTCF motifs provided the initial clue, and subsequent genetic evidence using a DNA‐binding‐deficient PATZ1 mutant (C294A) established the causal necessity of this molecular mimicry. Crucially, co‐immunoprecipitation assays ruled out a stable protein–protein interaction, focusing the mechanism squarely on competition for the chromatin template. This “architectural competition” model represents a distinct layer of epigenetic dysregulation, wherein the genome's structural integrity is compromised not by breaking the architectural complex, but by displacing its key organizer (CTCF) through a “molecular mimic” that engages the same genomic coordinates to opposite functional ends. This expands our understanding of how cancer cells hijack the genome's structural machinery, offering a new perspective on oncogenic rewiring.

Our findings on the downstream axis provide a coherent and mechanistically grounded explanation for PATZ1's oncogenicity. The identification of ZBTB20 as the key node directly links the upstream epigenetic perturbation to a potent tumor‐suppressive function. The combination of CUT&Tag visualization, 3C‐based structural analysis, and genetic rescue experiments provides a compelling chain of evidence: PATZ1 competes with CTCF, collapses a specific chromatin loop, and thereby epigenetically silences ZBTB20. The subsequent discovery that ZBTB20 transcriptionally represses PMEPA1—a known negative feedback regulator of TGF‐β that can activate non‐canonical MAPK pathways—creates a logically sound and potent oncogenic cascade. The activation of p38 and its downstream target STAT1 upon ZBTB20 loss directly links this axis to well‐established pro‐tumorigenic processes. This seamless connection from chromatin conformation to signal transduction underscores the robustness and biological relevance of the identified pathway.

Our study fills several critical knowledge gaps in the field. First, it is the first to define a comprehensive oncogenic role and mechanism for PATZ1 in melanoma, a function conserved across BRAF‐ and NRAS‐mutant genetic backgrounds. Second, it reveals a novel mechanism of CTCF functional interference via competitive DNA binding, a concept with likely broad implications beyond melanoma. Third, it is the first to define the tumor‐suppressive function of ZBTB20 in melanoma and to elucidate that its loss is a consequence of PATZ1‐mediated disruption of a specific chromatin loop, rather than genetic mutation. Finally, by integrating the PMEPA1‐p38‐STAT1 axis, we provide a complete mechanistic circuit from epigenetic alteration to malignant phenotype.

The clinical implications of our work are substantial. The PATZ1‐high/ZBTB20‐low expression signature we identified holds immediate promise as a powerful prognostic biomarker for melanoma patients. More importantly, our work unveils new therapeutic vulnerabilities. Targeting the PATZ1‐DNA interaction interface or developing strategies to disrupt PATZ1's DNA‐binding ability could represent a novel epigenetic therapy. Alternatively, pharmacological reactivation of ZBTB20 function or inhibition of the downstream PMEPA1‐p38‐STAT1 axis could offer viable strategies to short‐circuit this pathway in PATZ1‐driven melanomas. Furthermore, our exploratory upstream analysis, while preliminary, suggests additional therapeutic contexts. The association between PATZ1 amplification and its overexpression suggests that tumors with this genetic feature may be exquisitely dependent on this pathway. The strong correlation with MITF raises the intriguing possibility that the PATZ1/CTCF‐ZBTB20 axis may be embedded within the core melanocyte lineage program, potentially influencing differentiation state and therapeutic response. Interestingly, the predicted yet negatively correlated relationship with STAT3 hints at potential complex, context‐dependent regulation or feedback mechanisms within the melanoma transcriptional network, warranting further investigation.

Despite these advances, our study has limitations that point to future directions. The upstream drivers of PATZ1 overexpression in melanoma, while preliminarily explored through bioinformatic analyses identifying copy‐number alterations and a correlation with MITF, require definitive experimental validation. Determining whether MITF or other factors (including STAT3 under specific conditions) directly regulate PATZ1 transcription is a crucial next step. Furthermore, while our functional and 3C data robustly support the loop disruption model, the direct visualization of the disrupted chromatin loop at the ZBTB20 locus by high‐resolution 3C‐based techniques (e.g., HiChIP) in future studies would provide even more definitive structural evidence. It will also be crucial to investigate whether this competitive paradigm operates in other cancer types where PATZ1 is overexpressed and CTCF binds key tumor suppressor genes. Finally, the development and testing of targeted therapies against this axis will be the ultimate translation of our findings.

In summary, we have uncovered and mechanistically dissected the “PATZ1/CTCF‐ZBTB20‐PMEPA1‐p38‐STAT1” oncogenic axis. By introducing and rigorously proving the concept of “competitive DNA binding” as a means to subvert chromatin architecture, we provide a new framework for understanding how transcription factor competition can drive tumor progression. This work offers fresh diagnostic and therapeutic avenues for combating melanoma.

## 5 Conclusions

In conclusion, our study establishes the transcription factor PATZ1 as a master oncogenic driver in melanoma. We delineate a complete pathogenic axis wherein PATZ1, via its sequence‐specific DNA‐binding ability, promotes tumor progression by competitively disrupting CTCF‐mediated chromatin architecture to epigenetically silence the tumor suppressor ZBTB20, thereby unleashing the PMEPA1‐p38‐STAT1 oncogenic signaling cascade. This “architectural competition” mechanism, directly validated through structural and genetic approaches, is operative across major melanoma genetic subtypes. Our work not only reveals the disruption of the PATZ1/CTCF balance as a novel layer of epigenetic dysregulation in cancer but also identifies the PATZ1‐ZBTB20 hub as an immediate prognostic biomarker and a promising source of therapeutic targets. The upstream association of PATZ1 with lineage‐specific and genomic amplification cues further informs its context‐specific dysregulation. Ultimately, this research opens new avenues for combating this aggressive malignancy by targeting the vulnerability of transcription factor‐mediated competitive chromatin regulation.

## Funding

This work was supported by the General Project of Major Research Plan for Social Development of the Shaanxi province (No. 2024SF‐YBXM‐295) and the Basic Research Program for Natural Science of Shaanxi Province (No. 2025JC‐YBQN‐1251).

## Ethics Statement

The Project Was Approved by the Biomedical Ethics Committee of Health Science Center of Xi'an Jiaotong University (XJTUAE2024‐2373) and the Medical Ethics Committee of the First Affiliated Hospital of Xi'an Jiaotong University (LLSBPJ‐2023‐356).

## Consent

Every individual patient who participated in the research signed a written informed consent.

## Conflicts of Interest

The authors declare no conflicts of interest.

## Supporting information




**Supporting File**: advs74571‐sup‐0001‐SuppMat.docx.

## Data Availability

The data that support the findings of this study are available from the corresponding author upon reasonable request.
