# Supplementary material for: Dysregulation of the PATZ1/CTCF Balance Silences ZBTB20 to Drive Melanoma Progression
Source: Adv Sci (Weinh). 2026 Feb 25;13(26):e20917. doi: 10.1002/advs.202520917 (PMC13159142; doi:10.1002/advs.202520917)
Supplement: Supplementary file 1 — Supporting File: advs74571‐sup‐0001‐SuppMat.docx. [file ADVS-13-e20917-s001.docx]

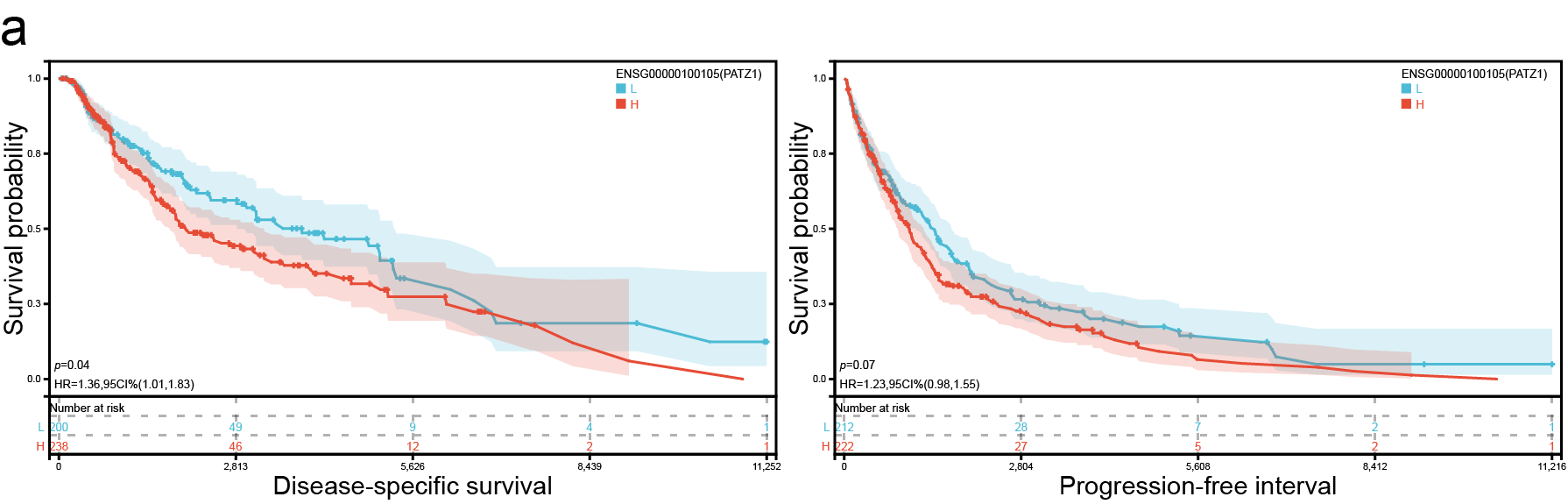


**Figure S1**. PATZ1 expression is associated with poor prognosis in melanoma.
(a) Kaplan-Meier analysis of disease-specific survival (DSS) and progression-free interval (PFI) for melanoma patients with high vs. low PATZ1 expression (TCGA data).


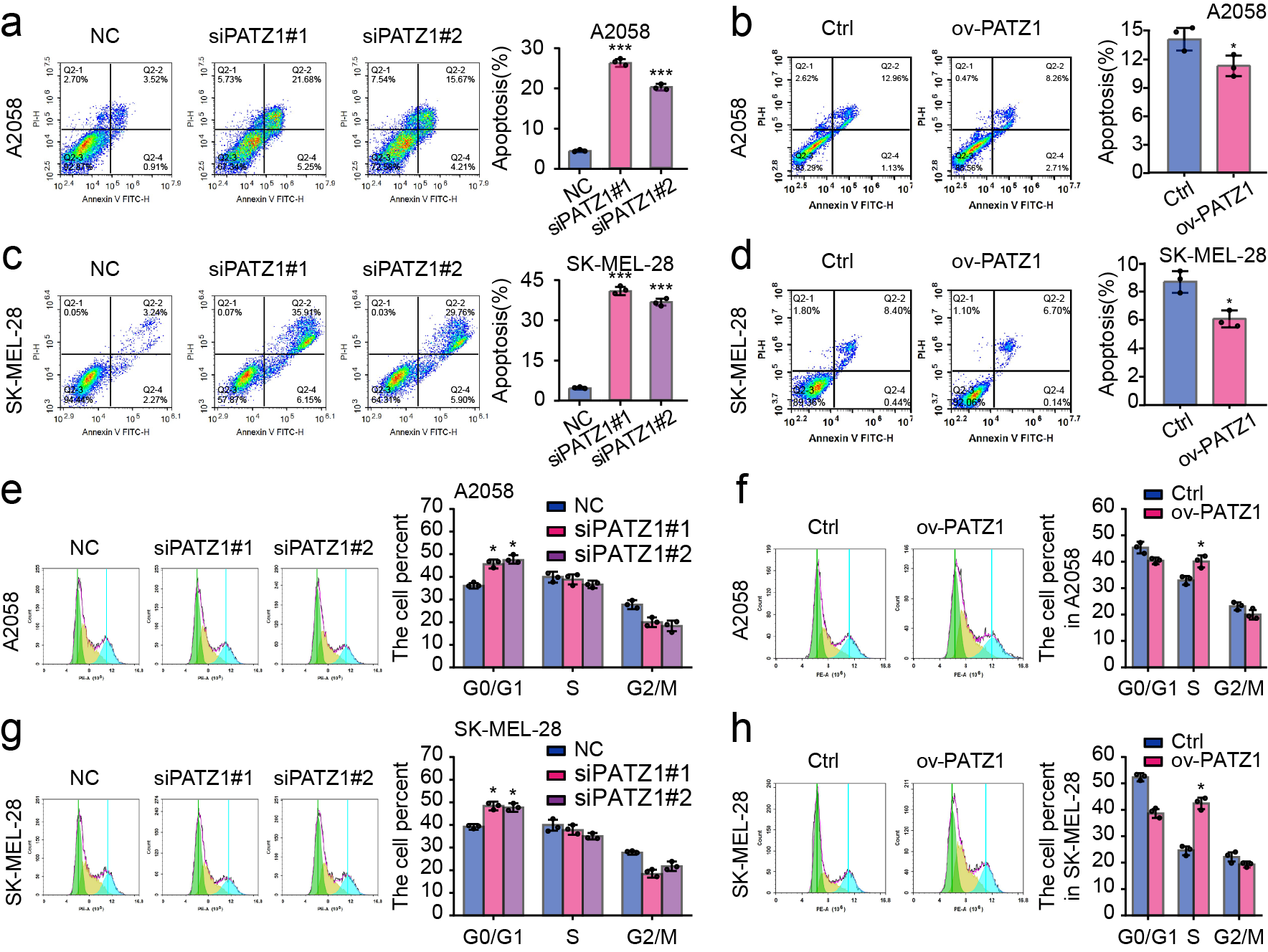


**Figure S2**. PATZ1 regulates melanoma cell apoptosis and cell cycle progression.
(a-d) Flow cytometry analysis of apoptosis in A2058 and SK-MEL-28 cells upon PATZ1 knockdown (a, b) and overexpression (c, d). (e-h) Flow cytometry analysis of cell cycle distribution in A2058 and SK-MEL-28 cells upon PATZ1 knockdown (e, f) and overexpression (g, h). Each experiment was performed in triplicate. According to the data characteristics, quantitative data of (a-h) were analyzed by Student’s t-test, **P* < 0.05, ***P* < 0.01, ****P* < 0.001.


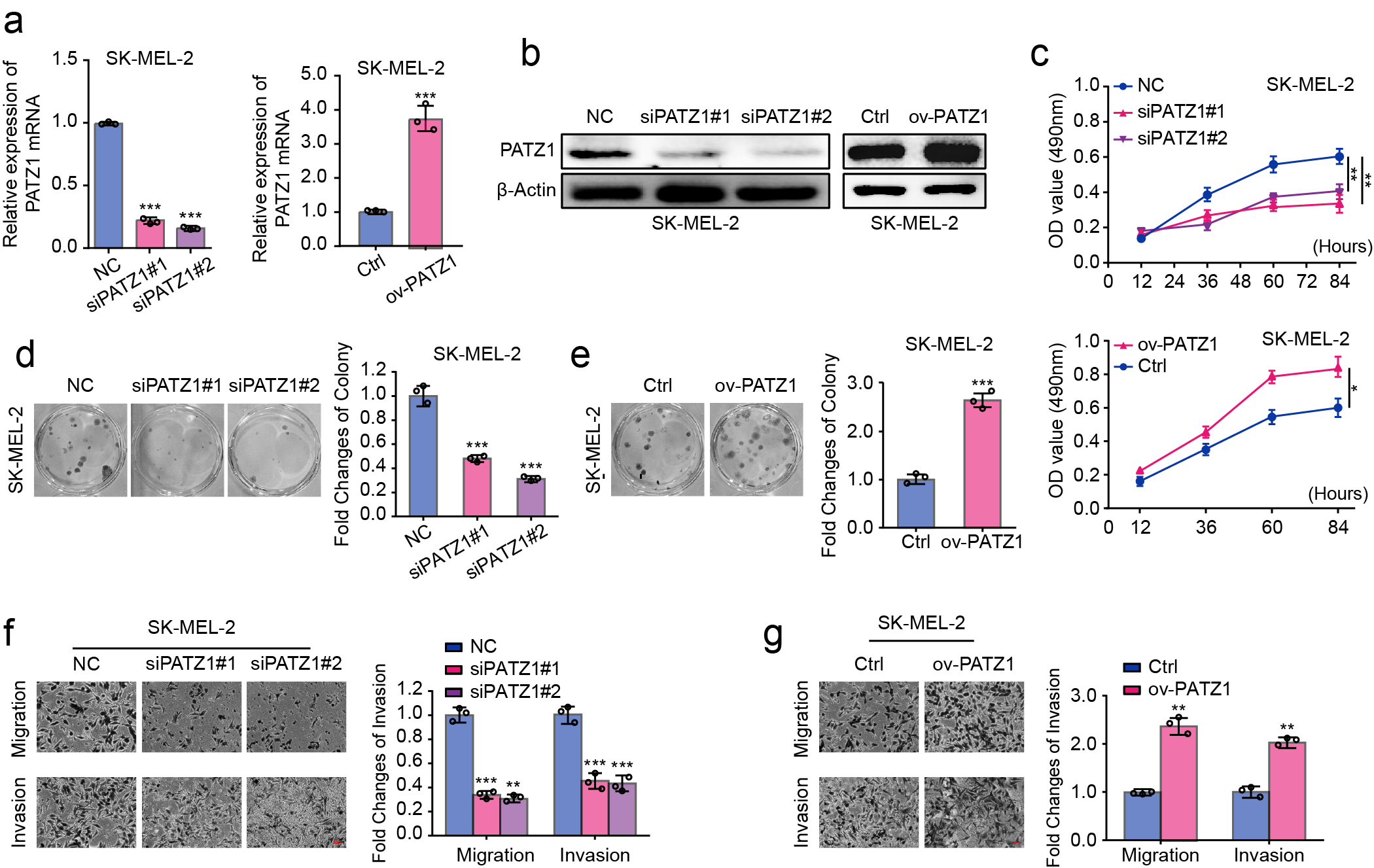


**Figure S3**. Oncogenic role of PATZ1 is conserved in NRAS-mutant melanoma
(a) qRT-PCR analysis confirming the efficiency of PATZ1 knockdown and overexpression in SK-MEL-2 cells. GAPDH was used as an internal reference. (b) western blot analysis confirming the efficiency of PATZ1 knockdown and overexpression in SK-MEL-2 cells. β-Actin was used as an internal reference.(c) Cell viability assessed by MTT assay upon PATZ1 knockdown and overexpression. (d, e) Clonogenic ability of melanoma cells upon PATZ1 knockdown (d) and overexpression (e). (f, g) Migratory and invasive capacities of melanoma cells evaluated by Transwell assays upon PATZ1 knockdown (f) and overexpression (g). Each experiment was performed in triplicate. According to the data characteristics, quantitative data of (a, d-g) were analyzed by Student’s t-test, quantitative data of (c) were analyzed by one-way ANOVA followed by Tukey’s post-hoc test for multiple comparisons, **P* < 0.05, ***P* < 0.01, ****P* < 0.001.


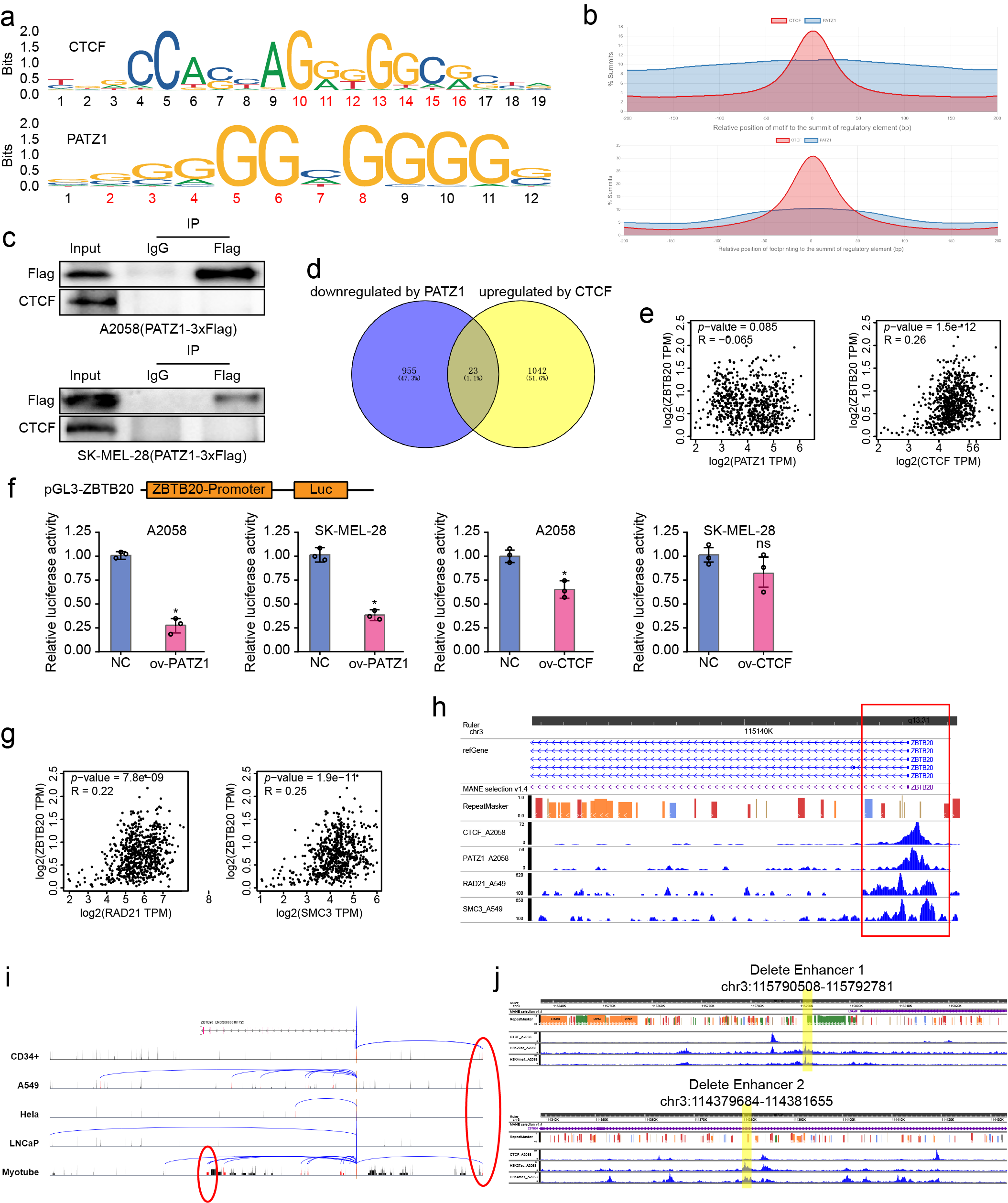


**Figure S4**. Bioinformatic and functional analysis of the PATZ1/CTCF-ZBTB20 axis.

(a) Sequence logos depicting the high similarity between the predicted DNA-binding motifs of human PATZ1 and CTCF (JASPAR database). (b) Genome-wide colocalization analysis of PATZ1 and CTCF binding sites using TFSyntax. (c) Co-immunoprecipitation assay in A2058 cells overexpressing Flag-tagged PATZ1 (PATZ1-Flag) or empty vector. Immunoprecipitation was performed with anti-Flag beads, and blots were probed for Flag and endogenous CTCF. Input represents 2% of lysate. (d) A Venn diagram illustrating the integrative analysis to identify downstream targets. The analysis intersected genes bound by PATZ1 (from PATZ1 CUT&Tag), repressed upon PATZ1 knockdown (from transcriptomic data), bound by CTCF (from CTCF CUT&Tag), and activated upon CTCF knockdown (from transcriptomic data), identifying ZBTB20 as a key candidate. (e) Correlation analysis of CTCF vs. ZBTB20 and PATZ1 vs. ZBTB20 mRNA expression in the TCGA-SKCM cohort (GEPIA2). Pearson’s r and P-value are shown. (f) Dual-luciferase reporter assay measuring the activity of the ZBTB20 promoter upon co-transfection with PATZ1 or CTCF overexpression plasmids in A2058 cells. (g) Correlation analysis of ZBTB20 expression with cohesin subunits SMC3 and RAD21 in the TCGA-SKCM cohort (GEPIA2). (h) Public ChIP-seq data tracks (ENCODE) showing binding of cohesin subunits (SMC3, RAD21) and active histone marks (H3K4me3, H3K9ac) at the ZBTB20 promoter region in human cell lines. (i) Schematic diagram of the strategy for predicting enhancers at the ZBTB20 locus using EnhancerAtlas. (j) Schematic representation of the CRISPR/Cas9 strategy for deleting Enhancer1 and Enhancer2. Each experiment was performed in triplicate. According to the data characteristics, quantitative data of (f) were analyzed by Student’s t-test, **P* < 0.05, ***P* < 0.01, ****P* < 0.001; ns, not significant.


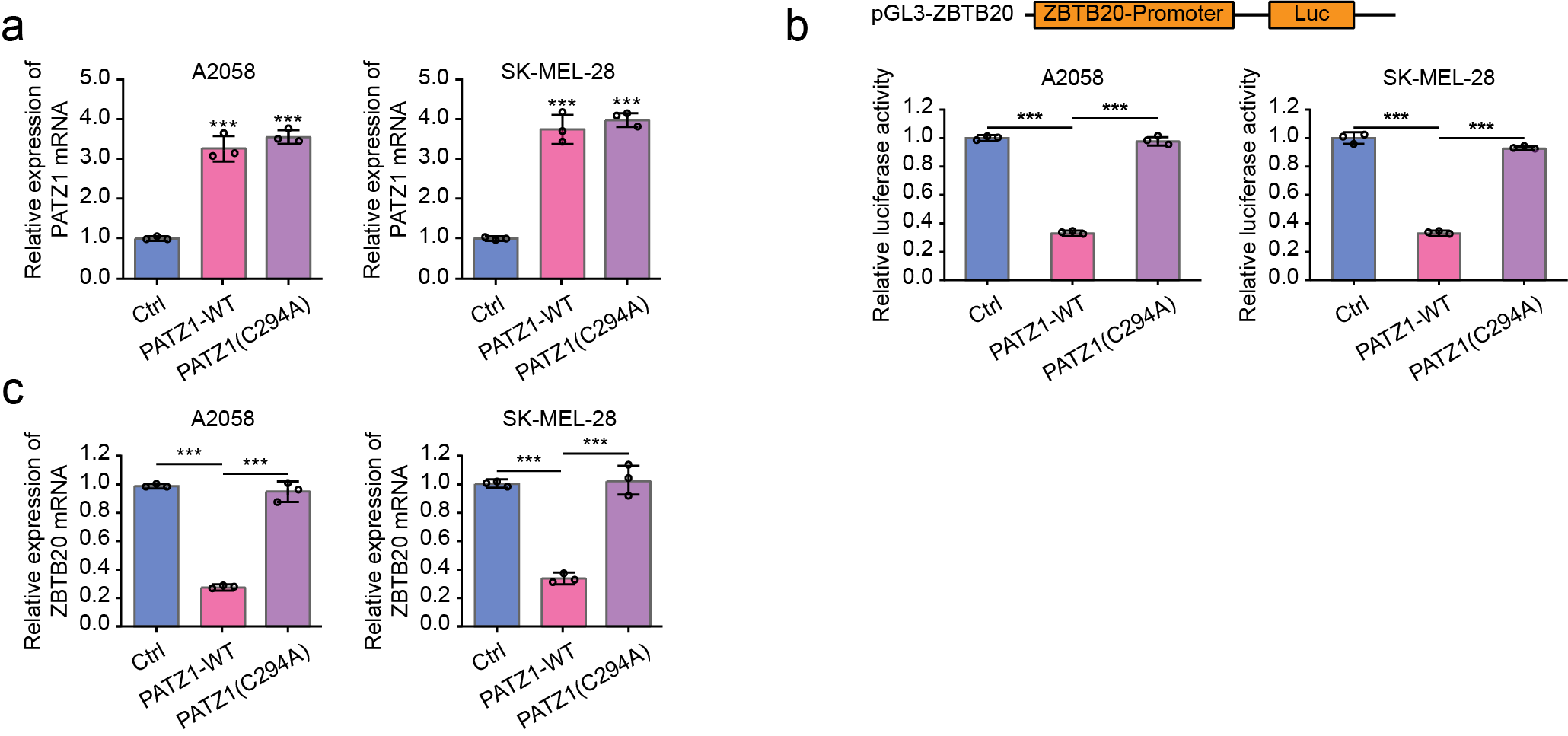


**Figure S5**. Functional characterization of the PATZ1 DNA-binding mutant. (a) qRT-PCR analysis confirm comparable PATZ1 mRNA expression levels of wild-type PATZ1 (PATZ1-WT) and the PATZ1(C294A) mutant in transfected A2058 cells. GAPDH was used as an internal reference. (b) Dual-luciferase reporter assay assessing the activity of the ZBTB20 promoter in A2058 cells co-transfected with PATZ1-WT or PATZ1(C294A) expression plasmids. Data are normalized to the empty vector control. (c) qRT-PCR analysis confirm comparable ZBTB20 mRNA expression levels of wild-type PATZ1 (PATZ1-WT) and the PATZ1(C294A) mutant in transfected A2058 cells. GAPDH was used as an internal reference. Each experiment was performed in triplicate. According to the data characteristics, quantitative data of (a-c) were analyzed by Student’s t-test, **P* < 0.05, ***P* < 0.01, ****P* < 0.001.


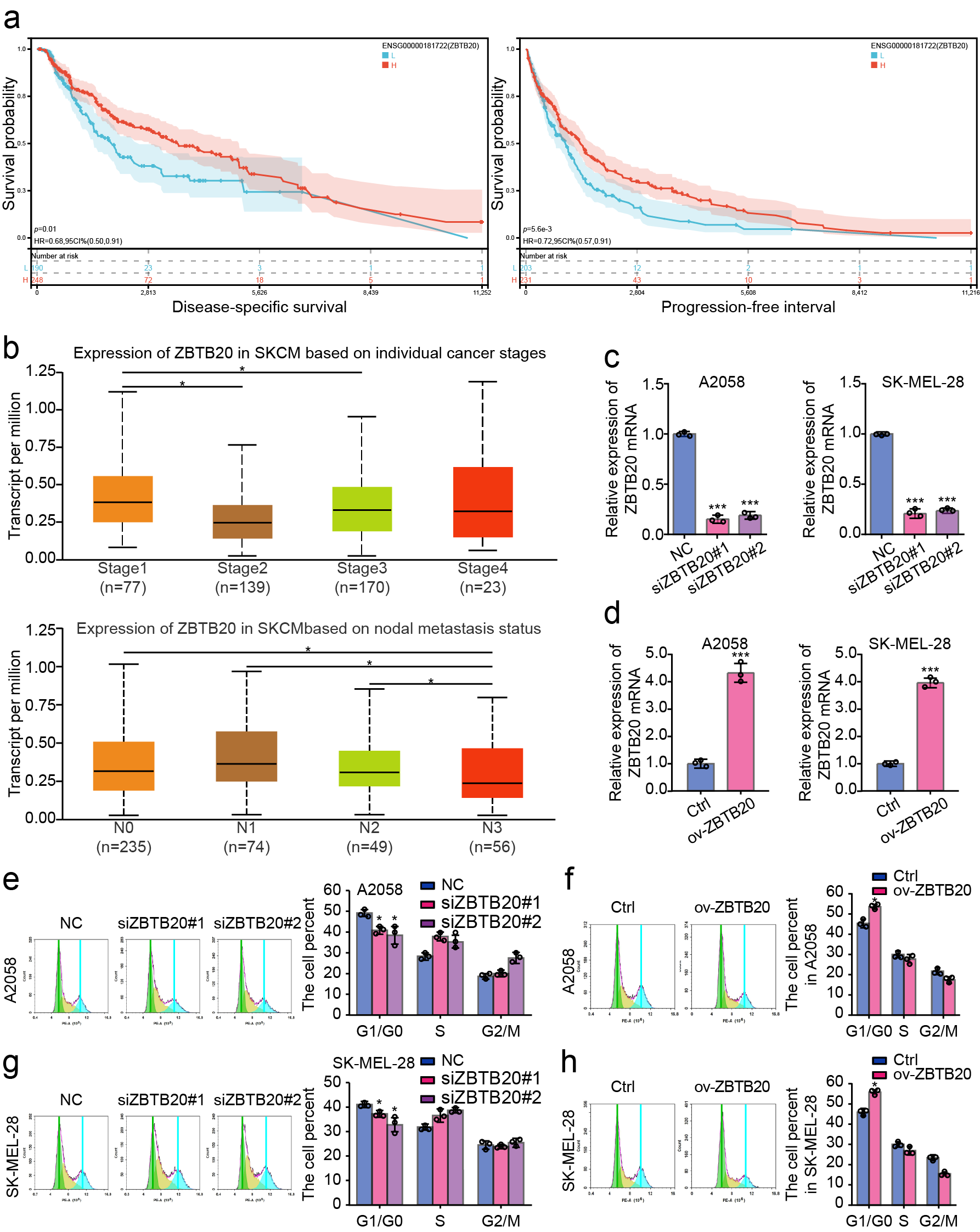


**Figure S6**. Clinical relevance and functional validation of ZBTB20 in melanoma.

(a) Kaplan-Meier analysis of disease-specific survival (DSS) and progression-free interval (PFI) for melanoma patients stratified by ZBTB20 expression. (b) Analysis of ZBTB20 expression correlation with clinical stages and nodal metastasis status from the UALCAN database. (c, d) qRT-PCR analysis confirming the efficiency of ZBTB20 knockdown (c) and overexpression (d). GAPDH was used as an internal reference. (e-h) Flow cytometry analysis of cell cycle distribution upon ZBTB20 knockdown (e, f) and overexpression (g, h). Each experiment was performed in triplicate. According to the data characteristics, quantitative data of (b-h) were analyzed by Student’s t-test, **P* < 0.05, ***P* < 0.01, ****P* < 0.001.


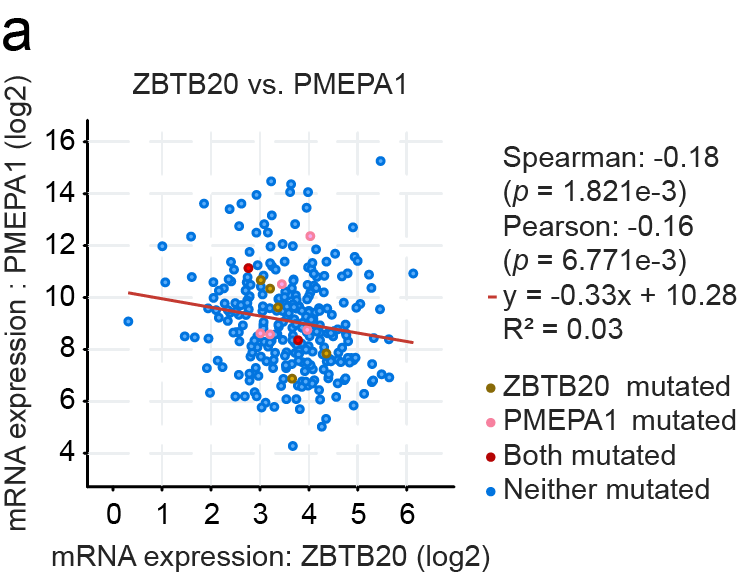


**Figure S7**. ZBTB20 transcriptionally represses PMEPA1.
(a) Analysis of the negative correlation between ZBTB20 and PMEPA1 mRNA expression in melanoma cohorts from cBioPortal.


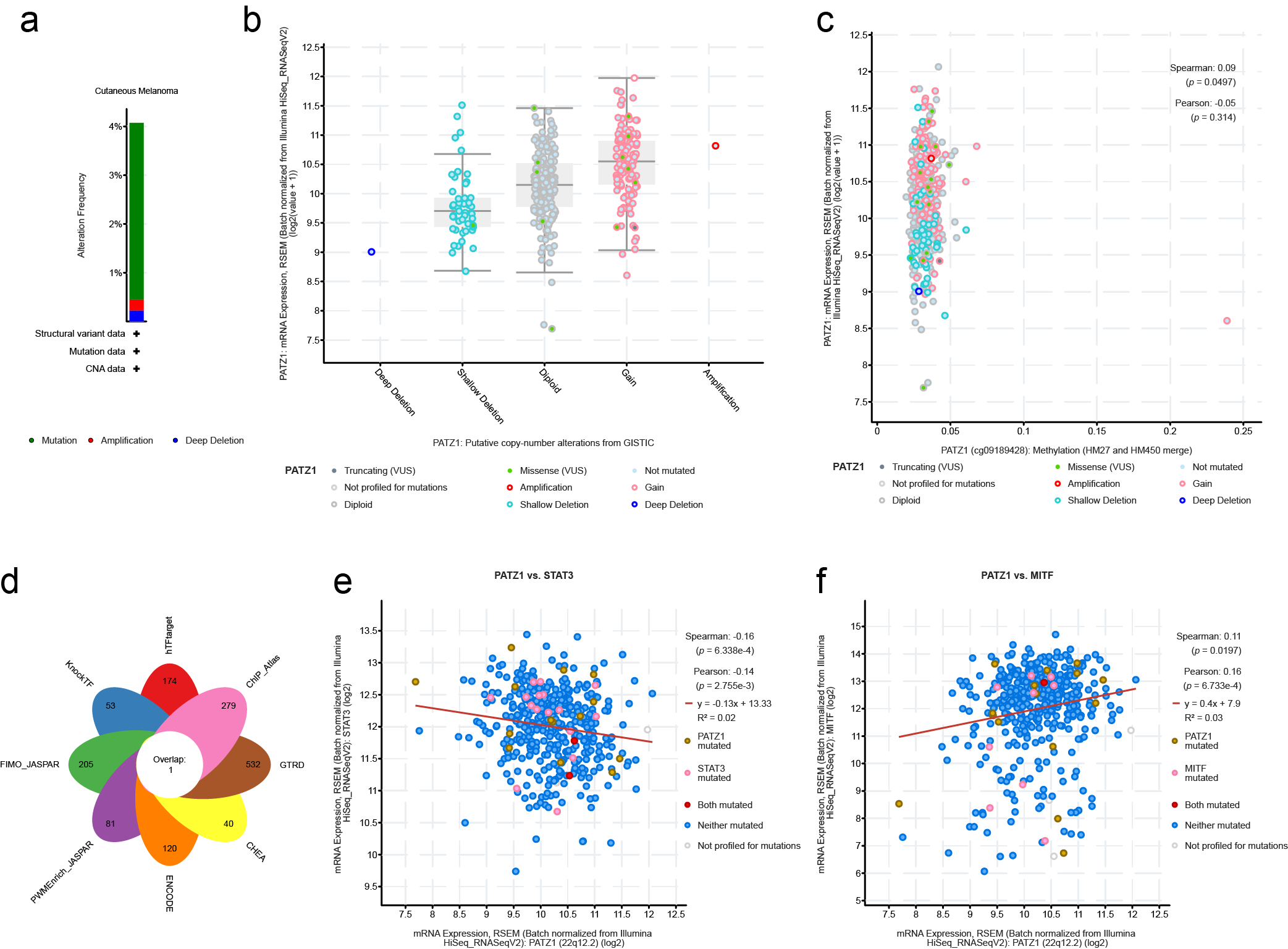


**Figure S8**. Exploratory Analysis of Potential Upstream Drivers of PATZ1 Overexpression
(a) Frequency of genomic alterations at the PATZ1 locus in the TCGA Skin Cutaneous Melanoma (SKCM) cohort, as analyzed via cBioPortal. “Amplification” and “Gain” represent copy-number alterations. (b) Correlation between PATZ1 copy-number variation (linear CNA value from cBioPortal) and its mRNA expression level (RNA-seq RSEM) in the TCGA-SKCM cohort. (c) Analysis of the relationship between DNA methylation (beta value) at the PATZ1 promoter CpG island (cg13625763) and PATZ1 mRNA expression in the TCGA-SKCM cohort. No significant inverse correlation was observed. Pearson’s r and P-value are shown. (d) Schematic summarizing the prediction of upstream transcriptional regulators of PATZ1 by integrating results from the JASPAR, GTRD, CHEA, KnockTF, ChIP_Atlas, hTFtarget, and ENCODE databases. STAT3 was identified as a candidate. (e) Correlation analysis between STAT3 and PATZ1 mRNA expression in the TCGA-SKCM cohort (GEPIA2). A significant negative correlation was observed. Pearson’s r and P-value are shown. (f) Correlation analysis between MITF and PATZ1 mRNA expression in the TCGA-SKCM cohort (GEPIA2). A significant positive correlation was observed. Pearson’s r and P-value are shown. Data in b, c, e, and f are derived from the indicated public cohorts. Statistical significance of correlations was assessed using Pearson’s correlation test.
